# Proximity proteomics provides a new resource for exploring the function of Afadin and the complexity of cell-cell adherens junctions

**DOI:** 10.1242/bio.061811

**Published:** 2025-01-30

**Authors:** Wangsun Choi, Dennis Goldfarb, Feng Yan, Michael B. Major, Alan S. Fanning, Mark Peifer

**Affiliations:** ^1^ Department of Biology, University of North Carolina at Chapel Hill, CB#3280, Chapel Hill, NC 27599-3280, USA; ^2^ Department of Cell Biology and Physiology, Washington University School of Medicine, St. Louis, MO, USA 63110; ^3^ Institute for Informatics, Data Science & Biostatistics, Washington University School of Medicine, St. Louis, MO, USA 63110; ^4^ Lineberger Comprehensive Cancer Center, University of North Carolina at Chapel Hill, Chapel Hill, NC 27599, USA; ^5^ Department of Cell Biology and Physiology, University of North Carolina at Chapel Hill, Chapel Hill, NC 27599, USA

**Keywords:** Afadin, Cell adhesion, Adherens junctions, Canoe, BioID

## Abstract

The network of proteins at the interface between cell-cell adherens junctions and the actomyosin cytoskeleton provides robust yet dynamic connections that facilitate cell shape change and motility. While this was initially thought to be a simple linear connection via classic cadherins and their associated catenins, we now have come to appreciate that many more proteins are involved, providing robustness and mechanosensitivity. Defining the full set of proteins in this network remains a key objective in our field. Proximity proteomics provides a means to define these networks. Mammalian Afadin and its *Drosophila* homolog Canoe are key parts of this protein network, facilitating diverse cell shape changes during gastrulation and other events of embryonic morphogenesis. Here we report results of several proximity proteomics screens, defining proteins in the neighborhood of both the N- and C-termini of mammalian Afadin in the premier epithelial model, MDCK cells. We compare our results with previous screens done in other cell types, and with proximity proteomics efforts with other junctional proteins. These reveal the value of multiple screens in defining the full network of neighbors and offer interesting insights into the overlap in protein composition between different epithelial cell junctions.

## Teaser

**Summary:** Afadin BioID reveals new candidate adherens junction proteins, opening leads for new research and suggesting overlap with other cell junctions.

## INTRODUCTION

The multiprotein complexes at cell-cell and cell-extracellular matrix junctions establish and maintain epithelial tissue architecture and facilitate cell shape changes and cell migration during both morphogenesis and tissue homeostasis (Perez-Vale and Peifer, 2020). To accomplish these tasks, junctional protein complexes need to be connected to the contractile actomyosin cytoskeleton (Cronin and DeMali, 2021). Defining the full set of molecular components at each junction and unraveling their collective functions is a key challenge for our field.

Work over several decades revealed that cell-extracellular matrix junctions link to the cytoskeleton via a complex, layered network of dozens of proteins (Case and Waterman, 2015). In contrast, until the mid-2000s, the view of cytoskeletal connections at cell-cell adherens junctions (AJs) was much simpler, suggesting direct linkage (Gates and Peifer, 2005). Transmembrane classic cadherins linked cells to one another by homophilic interactions. Beta-catenin bound both the cadherin cytoplasmic tail and alpha-catenin, and alpha-catenin then bound actin.

However, this simplistic picture of AJ:cytoskeletal linkage has been replaced by one that is more complex in several ways. First, it is now clear that a large network of proteins is localized to AJs. Many are large, multidomain proteins that interact with one another by complex, multivalent linkages (Rouaud et al., 2020). Second, AJs are mechanosensitive and mechanoresponsive (Buckley et al., 2014; Campàs et al., 2024; Wang et al., 2022), with conformational change and protein recruitment strengthening cytoskeletal connections when junctions are under mechanical tension (Yap et al., 2018). This revealed many additional proteins as part of the network of proteins linking AJs to the cytoskeleton (Perez-Vale and Peifer, 2020), including p120, Par3, Afadin/Canoe, ZO-1 family members, vinculin, and Ajuba. In addition to actin and non-muscle myosin, additional cytoskeletal regulators are also enriched at AJs, including Eva/VASP proteins (Gates et al., 2007), and at least in some cell types, Diaphanous-class formins (Homem and Peifer, 2008) and the Arp2/3 complex (Kovacs et al., 2002). Signaling proteins also localize to AJs, including both transmembrane receptors like the epidermal growth factor receptor (Fedor-Chaiken et al., 2003) and cytoplasmic signaling molecules, like non-receptor tyrosine kinases in the Src (Calautti et al., 1998) and Abl families (Stevens et al., 2008).

Cell-cell AJs also need to be dynamic, so that they can be rapidly assembled and disassembled, both during major changes in cell adhesion such as those occurring during epithelial mesenchymal transitions and their reversal, and the adjustments that need to be made as cells change chape and move or adjust to challenges such as mitosis or extrusion. This involves many additional levels of regulation, including initial assembly as cells form new junctions (Harris, 2012), trafficking via membrane delivery, endocytosis, and recycling (Bruser and Bogdan, 2017) (West and Harris, 2016), and cis- and trans-interactions between cadherins (Troyanovsky, 2022).

AJs are only one part of what is often referred to as the apical junctional complex. In vertebrate cells tight junctions (TJs) assemble just apical to AJs, where they provide epithelial barrier function. Strands of transmembrane claudins link cells together, thus assembling the barrier to diffusion between cells, and, like cadherins, claudins are linked to a complex network of peripheral membrane proteins that link claudins to one another and link TJs to the actin cytoskeleton (Rouaud et al., 2020). Key among these are scaffolding proteins in the ZO-1 family. Intriguingly, ZO-1 proteins also localize to AJs as they assemble and are important for their conversion to mature junctions (Ikenouchi et al., 2007). ZO-1 family proteins also bind the AJ proteins alpha-catenin and Afadin (Itoh et al., 1997; Takahashi et al., 1998; Yokoyama et al., 2001). These data and others reveal crosstalk between AJs and TJs (Rouaud et al., 2020). Specialized proteins are enriched at tricellular junctions where three cells meet, including vertebrate tricellulin, angulin-1/LSR, angulin-2/ILDR1, and angulin-3/ILDR2, which localize at the level of TJs. These need to link to the core cadherin-catenin complex, and provide another example of AJ/TJ crosstalk (van den Goor and Miller, 2022). *Drosophila* also has proteins enriched at tricellular junctions, Anakonda, Gliotactin, Sidekick and M6 proteins, but these localize at the level of AJs (Higashi and Chiba, 2020), as the claudin-containing junctions that provide barrier function in *Drosophila*, the septate junctions, are basal to AJs (Izumi and Furuse, 2014).

Several other classes of cell junctions also play important roles. The apical most region of the lateral membrane of both vertebrate and *Drosophila* cells is defined by the Crumbs complex (Buckley and St Johnston, 2022; Tan et al., 2020). Recent work reveals crosstalk between this apical complex and TJs, positioning the TJ apically (Pombo-Garcia et al., 2024). In vertebrate cells, lateral contacts are reinforced by the desmosomes, which are built around desmosomal cadherins and their cytoplasmic partners, which link to the intermediate filament cytoskeleton (Perl et al., 2024). There also is clear crosstalk between AJs and desmosomes, via both mechanical and chemical signals (Rubsam et al., 2018). Finally, integrin-based focal adhesions (FAs) link cells to the underlying extracellular matrix, and via networks of linker proteins can connect to both the actin and intermediate filaments cytoskeletons (Case and Waterman, 2015). One mechanism of crosstalk is that different junctions share overlapping set of linker proteins (Bachir et al., 2017) such as vinculin which is shared between AJs and FAs.

We focus on the roles of Afadin and its *Drosophila* homolog Canoe in AJ-cytoskeletal linkage. They play important roles in cell shape change and cell rearrangements during events ranging from initial positioning of cell-cell AJs (Choi et al., 2013) to the apical constriction and convergent elongation events of gastrulation (Ikeda et al., 1999; Sawyer et al., 2011, 2009; Zhadanov et al., 1999) to later collective cell migration (Boettner et al., 2003). Both also are important for the architecture of adult tissues, ranging from fly eyes (Gaengel and Mlodzik, 2003; Miyamoto et al., 1995) to mammalian kidneys (Yang et al., 2013). Unlike the cadherin-catenin complex, which localizes all along the lateral cell membrane, Afadin/Canoe is more tightly enriched at the apical end of the lateral interface, in the structure often referred to as the zonula adherens, though in at least one mammalian cell type, Afadin moves to an even more apical level as AJs mature. In this tissue it moves apically from a location mixed with cadherin-catenin complexes, but still remains basal to the tight junctions (Mangeol et al., 2024).

**Fig. 1.**
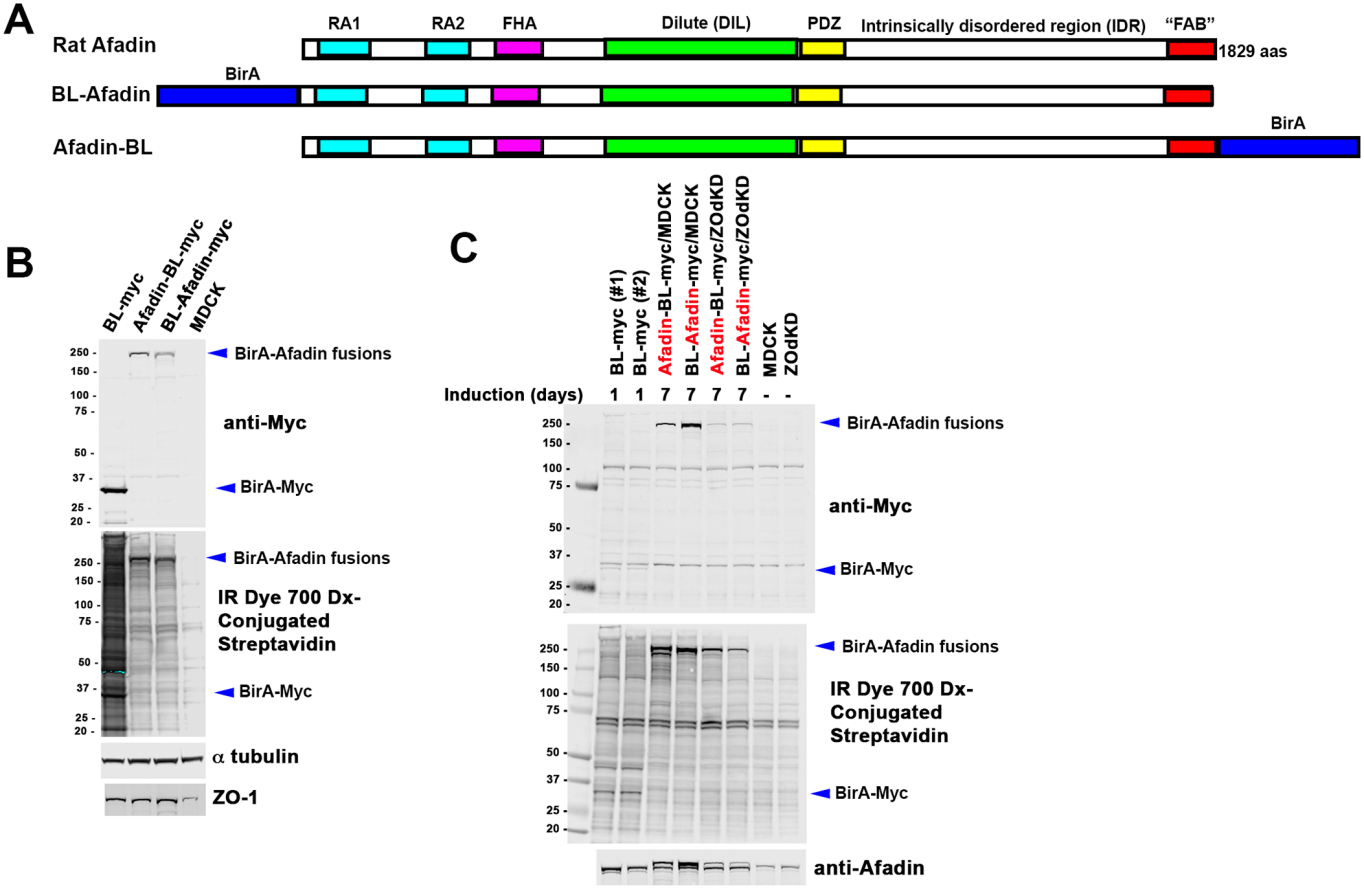
**Designing and validating reagents to identify potential Afadin protein partners and neighbors.** (A) Diagram of Afadin and the BirA*-fusions used. (B) Immunoblot of cell extracts from MDCK cells induced to express our BirA*-myc control, Afadin-BL-myc, BL-Afadin-myc or no construct. Top panel. Immunoblot with anti-myc antibody to detect expressed constructs. Middle panel, blot with IR Dye 700 Dx-Conjugated Streptavidin to detect biotinylated proteins. Tubulin serves as a loading control. (C) Immunoblot of cell extracts of MDCK cells or ZO-knockdown MDCK cells induced to express our BirA*-myc control, Afadin-BL-myc, BL-Afadin-myc or no construct. Induction time of the BirA*-myc control was only 1 day, while induction time for the others was 7 days. Top panel. Immunoblot with anti-myc antibody to detect expressed constructs. Middle panel, blot with IR Dye 700 Dx-Conjugated Streptavidin to detect biotinylated proteins. Bottom panel, immunoblot with anti-Afadin antibodies to confirm expression of tagged proteins.

Afadin/Canoe family members are found across the animal kingdom. They share a complex, multidomain protein architecture (Fig. 1A; Gurley et al., 2023; Smith, 2023). Most N-terminal are two RA domains, known to bind small GTPases in the Ras/Rap1 family. These GTPases use Afadin/Canoe as effectors, activating them upon binding. Next follow three additional conserved protein domains: a Forkhead-associated (FHA) domain, known in other proteins to bind phosphorylated peptides, a Dilute domain, only known from Afadin/Canoe and the non-conventional Myosin V family, and a PSD-95/discs large/zona occludens (PDZ) domain. This set of conserved protein domains is followed by a long intrinsically disordered region that includes a region or regions that bind filamentous actin. Afadin/Canoe's multidomain architecture allows it to interact with multiple proteins. These include small GTPases in the Ras/Rap1 family that bind the RA domains (McParland et al., 2024; Smith, 2023), the ADIP protein that can bind the Dilute domain (Asada et al., 2003), Scribble, which can bind the FHA domain (Goudreault et al., 2022), PLEKHA7, which can bind both RA and PDZ domains (Kurita et al., 2013), and the transmembrane junctional proteins Nectins (Takahashi et al., 1999), E-cadherin (Sawyer et al., 2009), Neurexin (Zhou et al., 2005), and JAM-A (Ebnet et al., 2000), which along with Eph family receptors (Buchert et al., 1999), the Notch ligand Jagged (Popovic et al., 2011), and the kinase/GEF/GAP Bcr (Radziwill et al., 2003) can bind the PDZ domain. Actin (Mandai et al., 1997), alpha-catenin (Pokutta et al., 2002), ZO-1 (Takahashi et al., 1998), Ponsin (Mandai et al., 1999), Lgn (Carminati et al., 2016), and profilin (Boettner et al., 2000) can bind to sites in the intrinsically disordered region. A recent cryo-EM structure revealed cooperative interactions between a conserved motif in the intrinsically disordered region that binds both actin and the actin-binding domain of alpha-catenin (Gong et al., 2024 preprint). Given this diversity of interaction domains and regions, it seems likely additional Afadin binding partners exist.

One key knowledge gap in our field is the full identity of the network of proteins connecting AJs to the cytoskeleton. Proteomics tools can help fill this gap. Proximity-labeling approaches like APEX or BioID offer the opportunity to take an assumption-free approach to identifying proteins in this network (Bosch et al., 2021). By fusing the protein of interest to an engineered enzyme that can add biotin to nearby proteins, and then purifying biotinylated proteins using streptavidin, one can identify both direct and indirect interactors (Roux et al., 2018). We used this approach to define and contrast proteins in the neighborhood of either the N- or C-termini of mammalian Afadin in one of the best-characterized of all mammalian epithelial cell lines, MDCK cells. We also examined the Afadin proximity protein is a second cell line – MDCK cells in which both ZO-1 and ZO-2 were knocked down (referred to below as ZOdKD cells). We included this cell line because in these cells junctional architecture is dramatically altered, with elevated levels of Afadin at cell junctions. and the assembly of a highly contractile sarcomeric actomyosin cytoskeleton at AJs (Choi et al., 2016). In these cells Afadin plays an important role in stabilizing the linkage of cell-cell adherens junctions to the contractile actomyosin cytoskeleton, as when Afadin is knocked down cell shapes are drastically altered and junctions pull apart from the cytoskeleton (Choi et al., 2016).

## RESULTS

### Designing and validating reagents to identify potential Afadin protein partners and neighbors

Afadin and its *Drosophila* homolog Canoe are conserved multidomain proteins that are key components in the network of proteins linking cadherin-based AJs to the actomyosin cytoskeleton. Some domains or regions have known binding partners, but the interactors for other well conserved domains remain unknown. We sought an unbiased approach to identify proteins within the Afadin protein network, including those binding directly and those that are proximal. To do so, we used BioID-based proximity labeling, in which the promiscuous biotin ligase enzyme (BirA-R118G, referred to as BirA*) is fused to a protein of interest. Following the addition of exogenous biotin to live cells expressing a BirA*:bait fusion, proteins within a ∼10-50 nm sphere are biotinylated. Biotinylated proximal proteins are then purified with streptavidin for downstream mass spectroscopy identification and quantification (Sears et al., 2019).

We previously cloned the coding sequence of mammalian Afadin tagged with both BirA* and a myc-epitope at either the N- or C-terminus (BL-Afadin or Afadin-BL) into doxycycline-regulated vectors (Fig. 1A; Bonello et al., 2019). In our previous analyses of Afadin function, we used the well-characterized epithelial cell line MDCK (Choi et al., 2016). We thus generated stable MDCK cell lines in which each fusion protein – BL-Afadin or Afadin-BL – was expressed after doxycycline-withdrawal (Bonello et al., 2019), alongside a control cell line expressing BirA* alone. We had also used MDCK cells in which both ZO-1 and ZO-2 were knocked down (referred to below as ZOdKD cells) – in these cells junctional architecture is dramatically altered, with elevated levels of Afadin and the assembly of a highly contractile sarcomeric actomyosin cytoskeleton at AJs (Choi et al., 2016). We thus also created stable lines for each fusion protein in this cell background.

We first assessed the expression of proteins, using antibodies to the myc-epitope. MDCK cells transfected with the BirA* construct alone, with BL-Afadin, or with Afadin-BL, were incubated in medium containing biotin. Immunoblotting revealed myc-tagged proteins of the expected sizes (Fig. 1B, top panel). In parallel, we transfected ZO-knockdown cells with the same constructs, and once again observed myc-tagged bands consistent with the sizes of BL-Afadin or Afadin-BL (Fig. 1C, top panel). Re-probing this blot with antibodies to Afadin confirmed these are myc-tagged Afadin, as we observed doublets of wild-type and tagged Afadin in cells in which our constructs were expressed (Fig. 1C, bottom panel). Finally, we re-probed these blots with IR Dye 700 Dx-Conjugated Streptavidin, which binds tightly to any biotinylated protein. We observed biotinylated proteins of the size of our constructs (Fig. 1B,C, middle panels), and also saw other biotinylated proteins. MDCK cells expressing BirA* alone had much higher levels of biotinylated proteins relative to the BL-Afadin or Afadin-BL cells, when cells were incubated for the same time (Fig. 1B, middle panel), and had similar levels of overall biotinylated protein when incubated with biotin for much shorter periods (1 versus 7 days; Fig. 1C, middle panel).

### BirA*-tagged versions of Afadin localize to cell-cell junctions

**Fig. 2.**
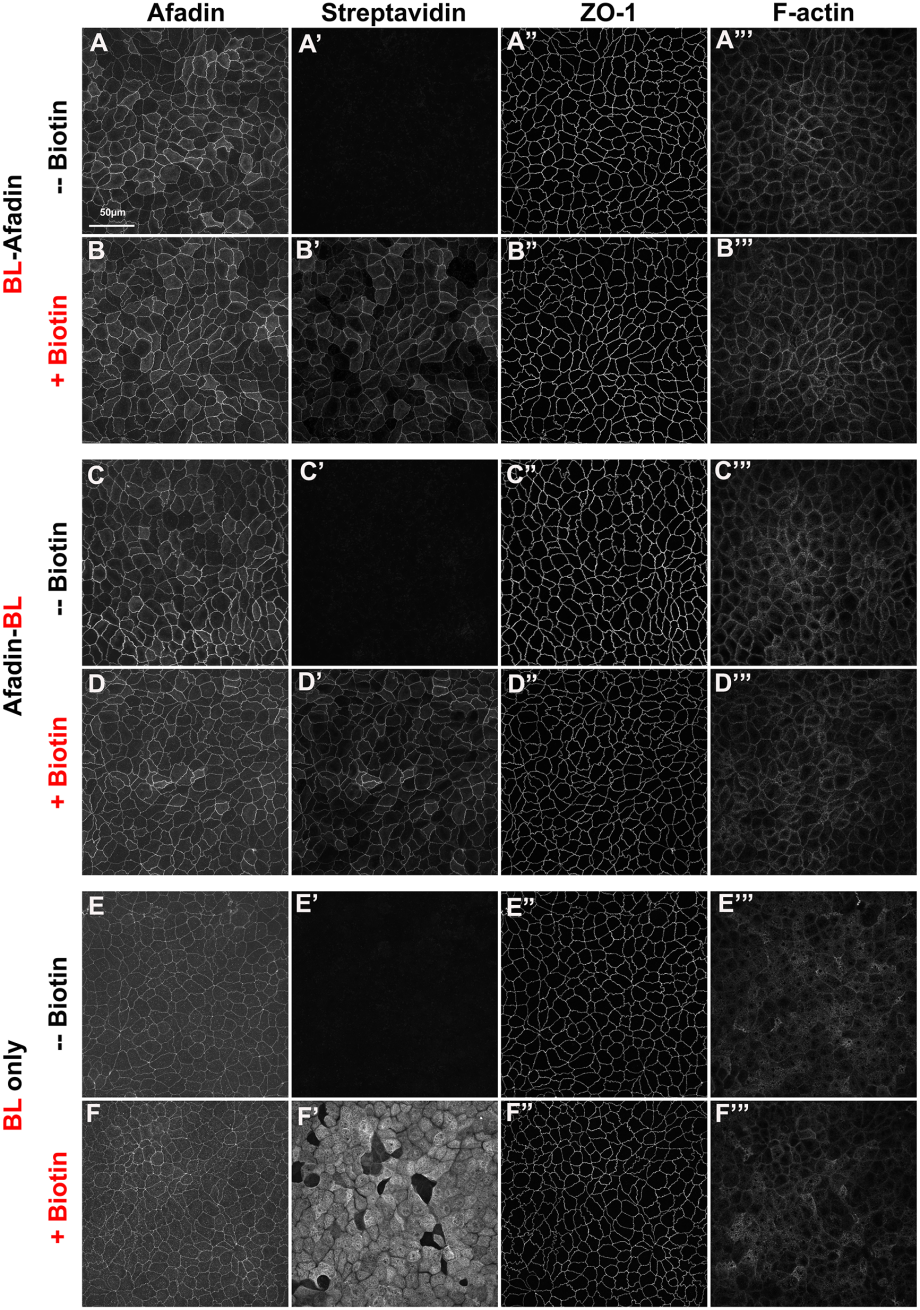
The BirA*-tagged versions of Afadin localize to cell junctions. MDCK cells cultured to confluence and prepared for immunofluorescence. Cells were cultured without or with added biotin in the medium. (A,B) Stable cell line expressing BL-Afadin. (C,D) Stable cell line expressing Afadin-BL. In both cases, cells with added biotin accumulate a streptavidin-labeled protein co-localizing with Afadin at cell junctions. (E,F) Stable cell line expressing our BirA*-myc control. In this case, addition of biotin leads to accumulation of streptavidin-labeled proteins throughout the cell.

To assess whether tagging altered protein localization, we examined whether the myc-tagged BirA*/Afadin fusions localized to cell-cell junctions, like wild-type Afadin. To do so, we immunostained cells with antibodies to Afadin, the junctional protein ZO-1, F-actin, and, in the same cells, visualized the localization of biotinylated proteins by immunostaining with an Alexa-568 conjugated streptavidin. Consistent with specificity, the streptavidin signal was highly enriched at cell-cell junctions and co-localized with BL-Afadin (Fig. 2B-B‴) or Afadin-BL (Fig. 2D-D‴). In contrast, in cells expressing BirA* alone, the streptavidin signal filled the whole cell, with no apparent enrichment at cell junctions (Fig. 2F-F‴). In each case, the streptavidin signal was dependent on addition of exogenous biotin (Fig. 2A′ versus B′,C′ versus D′,E′ versus F′).

### Some junctional proteins are biotinylated in cells expressing BirA*-tagged versions of Afadin

**Fig. 3.**
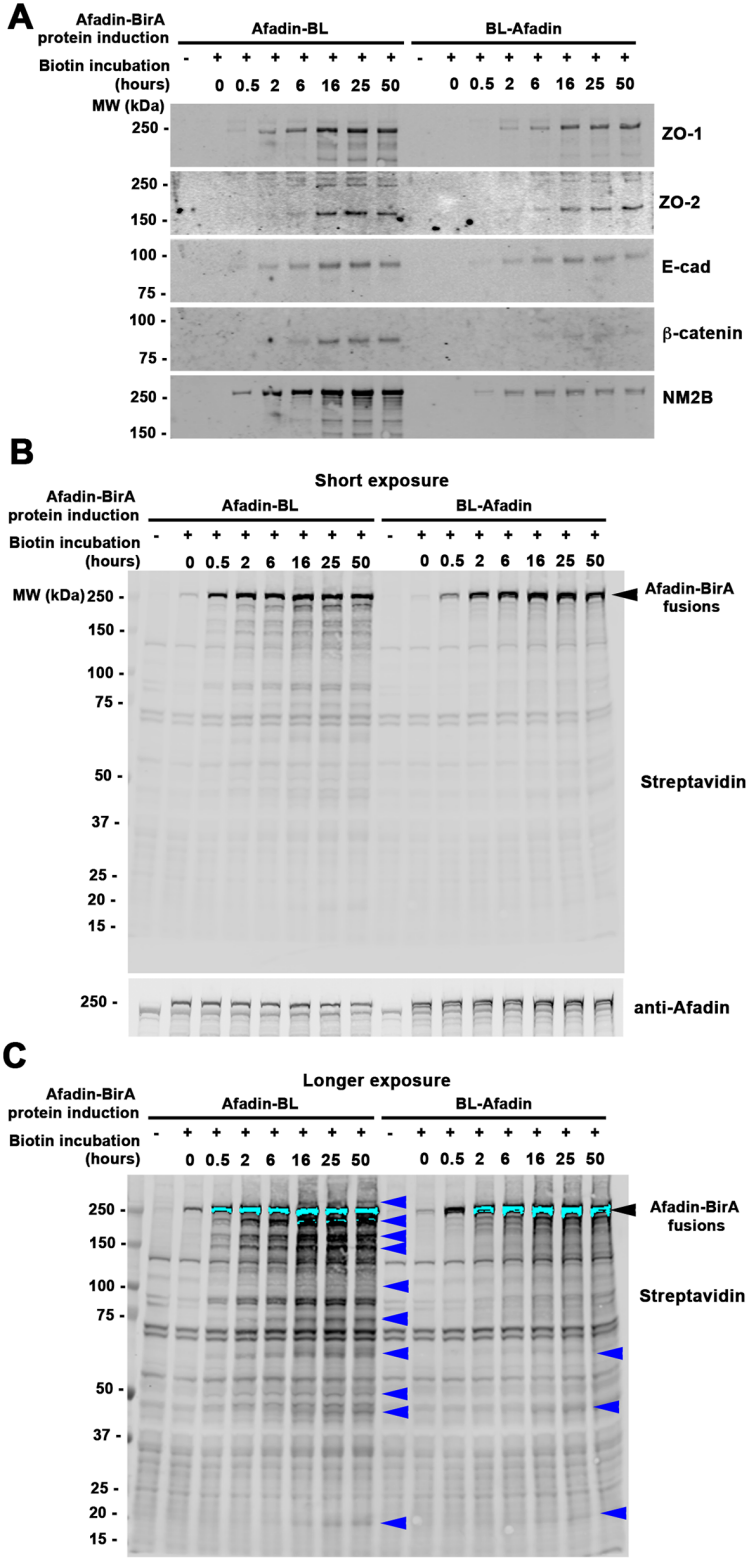
A subset of junctional proteins are biotinylated in cells expressing BirA*-tagged Afadin and a time course of accumulation of biotinylated proteins. (A) Stable MDCK cell lines carrying constructs expressing BirA*-tagged Afadin were cultured to confluence, exposed to biotin for varying amounts of time. Cell extracts were made, biotinylated proteins were affinity-purified from cells using streptavidin, analyzed by immunoblotting with antibodies to various junctional proteins. (B) Short exposure of an immunoblot of cell extracts from MDCK cells induced to express Afadin-BL-myc or BL-Afadin-myc, incubated with biotin over a time course of up to 50 h, and blotted with IR Dye 700 Dx-Conjugated Streptavidin to detect biotinylated proteins, or with Afadin to detect the expressed fusion. The BirA*-tagged constructs are seen at all time points, with a low level of self-biotinylation without biotin addition and increasing levels beginning at 0.5 h. (C) Long exposure of the immunoblot shown in B. Other biotinylated proteins begin to be seen accumulating as the time course progresses (blue arrows).

To further verify localization of our fusions to cell-cell junctions, and to begin to explore proteins that are proximal to Afadin, we examined selected junctional proteins. We incubated cells expressing either Afadin-BL or BL-Afadin in biotin over a time course of hours. We then affinity purified biotinylated proteins from cells using streptavidin before immunoblot analysis of selected junctional and cytoskeletal proteins (Fig. 3A). The tight junction proteins ZO-1 and ZO-2, known Afadin binding partners (Yamamoto et al., 1997), were readily recovered in the streptavidin pull down in cells expressing either Afadin-BirA* fusion (Fig. 3A, top two panels). We also recovered the core AJ proteins E-cadherin and beta-catenin (Fig. 3A, panels 3 and 4), though recovery of beta-catenin was weaker in cells expressing BL-Afadin. The heavy chain of cytoplasmic myosin was also detected (Fig. 3A, panel 5).

### Mass spectrometry-based proximity proteomic analysis of Afadin identified 144 proximal proteins in MDCK cells

A biotin time course immunoblot analysis revealed robust biotinylation of our tagged Afadin constructs after short incubation (Fig. 3B), with biotinylation of Afadin-proximal proteins increasing at longer times of biotin treatment (Fig. 3B versus C, blue arrows). Based on these data, we selected 24 h of biotinylation for unbiased mass spectrometry (MS) analysis. MDCK or ZOdKD cells expressing Afadin-BL, BL-Afadin, or the naked control BirA* fusion constructs were maintained in the presence of doxycycline to repress the expression of the transgenes (Tet-Off). For large-scale purification of biotinylated proteins, cells were cultured in 150 mm culture dishes in the presence of doxycycline until a monolayer formed (∼3-5 days). Once confluent, the culture was switched to doxycycline-free media to induce the fusion proteins and 50 µM biotin was added to the culture media for 24 h. Cell lysates were then used for subsequent streptavidin purification, protein digestion with trypsin and label-free liquid chromatography–tandem MS analysis.

Biological duplicate experiments were performed for MDCK cells expressing the control BirA*, Afadin-BL, and BL-Afadin. A single replicate of Afadin-BL and BL-Afadin in ZOdKD cells was analyzed. We first used the SAINT (significance analysis of interactome) express algorithm to identify statistically significant discoveries versus control using a cutoff of SAINT ≥0.9. The SAINT Score is a confidence score representing the strength of an interaction between a bait and a given prey protein. The score is a posterior probability of a true interaction and is calculated by comparing the observed Intensity for a Bait-Prey pair to probability distributions of true and false protein interactions modeled from the experimental and control data. A SAINT score above 0.9 represents a high probability of a true protein-protein interaction based on the data. The SAINT score also accounts for variance between replicates, and thus the high number of confident interactions demonstrates good reproducibility. Every proximal interaction that passed the SAINT threshold was observed in both replicates with similar spectral counts. We created a pairwise correlation plot which explicitly examines variance between replicates (Fig. S1). These data show that replicates are very similar, especially at higher abundance.

We then ordered the proteins on each list by the difference in average log2 Label Free Quantification (LFQ) intensity values between experimental and control (BirA* alone) groups. Doing so, we identified 144 Afadin proximal proteins. These included 95 proteins for BL-Afadin (Table 1; in each case excluding Afadin) and 106 proteins for Afadin-BL (Table 2). 55 proteins were shared on both lists (Table 3; full proteomics data are in Table S1). We also note in these tables the proteins for which we recovered a biotinylated peptide. While each protein should be biotinylated, the sites of biotinylation will vary. Not every lysine will be modified, and of the ones that are they can be at substoichiometric amounts compared to the unmodified version. Furthermore, biotinylation blocks trypsin cleavage, which results in longer peptides, potentially too long for reliable identification. Since we did not do a stringent elution off of the streptavidin beads, opting for on-beads digestions, the number of biotinylated peptides recovered is also expected to be extremely small. That said, the presence of a biotinylated peptide should give more confidence to the proximity interaction as it suggests that the biotinylation was abundant and thus the protein more likely to be proximal in large amounts.

**Table 1.** Validated interactors with BL-Afadin

Gene name	Protein name	AVE Log2 fold change versus control	SAINT probability	Biotinylated	Junctional protein	Cytoskeleton associated or regulating	Overlap with BIOID screen of Goudreault et al. (2022)	Overlap with BIOID screen of Go et al. (2021)
TJP1	**Tight junction protein 1 =ZO-1**	12.7780924	1	Yes	AJs/TJs (1)			Yes
Afdn	Afadin (Af-6)	12.1404095	1	Yes	AJs			
SORBS2	**Sorbin and SH3 domain containing 2**	11.5111165	1		AJs (2)	Yes		
KIAA1217	KIAA1217=skt (sickle tail)	10.2709289	1					Yes
TJP2	**Tight junction protein 2=ZO-2**	10.2573929	1		AJs/TJs (3)			Yes
ISG15	ISG15 ubiquitin like modifier	9.36776352	1					
LMO7	**LIM domain 7**	9.00209713	1	Yes	AJs (4)	Yes		
LZTS2 LAPSER1	Leucine zipper putative tumor suppressor 2	8.84869671	1	Yes			Yes	Yes
USP6NL	USP6 N-terminal like=RN-Tre	8.52154589	1	Yes			Yes	Yes
ERBIN	Erbb2 interacting protein	8.35129452	1	Yes	AJs (5)			Yes
TJP3	**Tight junction protein 3=ZO-3**	8.28795433	1		AJs/TJs (6)			
SCRIB	**Scribble planar cell polarity protein**	8.20864773	1	Yes	AJs (7)		Yes	Yes
LPP	LIM domain containing preferred translocation partner in lipoma zyxin family)	7.85889816	1		Ajs (8)			
EPS8L2	EPS8 like 2	7.77036905	1					
TP53BP2	**Tumor protein p53 binding protein 2=Aspp2**	7.75083828	1		AJs (9)		Yes	Yes
FRMD4B	FERM domain containing 4B	7.73575497	1					Yes
NCK1	Cytoplasmic protein adapter	7.61687851	1	Yes				Yes
CGN	Cingulin	7.58417034	1		TJs (10)			Yes
PARD3B	Par-3 family cell polarity regulator beta	7.43453312	1		TJs (11)			
RAP1A	**Small monomeric GTPase (EC 3.6.5.2)**	7.32741737	1					
SLC25A1	Citrate transport protein	7.3180933	1					
ABLIM1	Actin binding LIM protein 1	7.26043606	1			Yes		
CTNND1	Catenin delta 1=p120	7.248909	1		Ajs (12)		Yes	Yes
ABLIM3	Actin binding LIM protein family member 3	7.10746861	1	Yes	AJs (13)	Yes		
SORBS1	**Sorbin and SH3 domain containing 1 (CAP or Ponsin)**	7.0669136	0	Yes	AJs (14)			Yes
CD2AP	CD2 associated protein	7.03594112	1			Yes		
PLEKHA6	Pleckstrin homology domain containing A6	7.0032711	1		AJs (15)			
PLEKHA5	Pleckstrin homology domain containing A5	6.99893856	1		AJs (16)		Yes	Yes
DPYSL2	Dihydropyrimidinase-related protein 2=Crmp2	6.88928509	1			Yes		
CRK	Cytoplasmic adapter protein	6.86488152	1	Yes				
CTNNA1	**Catenin alpha 1**	6.83287907	1		AJs (17)		Yes	Yes
NECTIN3	**Nectin cell adhesion molecule 3**	6.77633381	0.99	Yes	Ajs (18)		Yes	
DBNL	Drebrin like=Abp1	6.75377321	0.99	Yes	AJs (19)	Yes		
SIPA1L1	Signal induced proliferation associated 1 like 1	6.73113155	1					Yes
LIMA1	LIM domain and actin binding 1=EPLIN	6.64982224	1		Ajs (20)	Yes		
SHROOM2	Shroom family member 2	6.54771423	1		TJs (21)	Yes		
KIAA1671	KIAA1671	6.49944592	1			Yes		Yes
CLMN	Calmin	6.38746357	1			Yes		
PDLIM7	PDZ and LIM domain protein 7=Enigma	6.38129902	1		TJs/FAs (22)	Yes		
SEPTIN9	Septin 9	6.36480618	1					
CTTN	Cortactin	6.34295654	1		AJs/Desmosomes (23)	Yes		
PPL	Periplakin	6.33712482	1		Desmosomes (24)			
LASP1	LIM and SH3 domain protein 1	6.27045441	1		AJs (25)			
KIAA1522	KIAA1522=NHSL3 (NHS like 3)	6.26501942	0.98					
SWAP70	Switching B cell complex subunit SWAP70	6.14587021	0.95	Yes		Yes		
LAD1	Ladinin 1	6.11666203	1			Yes		
DLG1	Discs large homolog 1	6.05070019	0.99		AJs (26)		Yes	Yes
NECTIN2	**Nectin cell adhesion molecule 2**	6.04839611	1		AJs (27)		Yes	Yes
LUZP1	Leucine zipper protein 1	6.03731728	1			Yes		
CRKL	CRK like proto-oncogene, adaptor protein	5.95023441	1	Yes				
DVL3	Dishevelled segment polarity protein 3	5.92189884	0.99		TJs (28)			
WDR11	WD repeat domain 11	5.90604591	0.98					
SHROOM3	Shroom family member 3	5.82347298	1		AJs (29)	Yes	Yes	Yes
PDLIM5	PDZ and LIM domain 5	5.77620983	1	Yes		Yes		
SH3RF1	E3 ubiquitin-protein ligase SH3RF1=POSH Plenty of SH3s	5.73868752	1					
SIRT2	NAD-dependent protein deacetylase (SIR2-like protein 2)	5.66047478	0.99					
ZYX	Zyxin	5.64910793	1	Yes	Ajs (30)			
FAM91A1	Family with sequence similarity 91 member A1	5.61380482	1	Yes				
VASP	Vasodilator stimulated phosphoprotein	5.60648823	1			Yes		
KANK2	KN motif and ankyrin repeat domain-containing protein 2	5.56093836	0.99					Yes
UTRN	Utrophin	5.49411583	1			Yes		Yes
VCPIP1	Ubiquitinyl hydrolase 1 (EC 3.4.19.12)	5.39645958	1	Yes				
SF1	Splicing factor 1	5.19843006	0.99					
EPB41L1	Erythrocyte membrane protein band 4.1 like 1=protein 4.1B	5.08696938	1			Yes		
PPP1CB	Serine/threonine-protein phosphatase (EC 3.1.3.16)	5.07891178	0.99					
COBLL1	Cordon-bleu WH2 repeat protein like 1	4.92169094	1			Yes		
OAS1	2'-5' oligoadenylate synthase (EC 2.7.7.84)	4.87281036	0.99					
EPB41	Erythrocyte membrane protein band 4.1	4.81721783	0.99		TJs (31)	Yes		
PFKL	ATP-dependent 6-phosphofructokinase (ATP-PFK)	4.73240185	0.99					
EIF4ENIF1	Eukaryotic translation initiation factor 4E nuclear import factor 1	4.70754004	0.99					Yes
USP7	Ubiquitin carboxyl-terminal hydrolase 7 (EC 3.4.19.12)	4.63778687	0.99					
RASSF8	Ras association domain family member 8	4.61238575	0.99		AJs (32)		Yes	Yes
STAT3	Signal transducer and activator of transcription	4.5264473	1					
CORO1C	Coronin	4.48379993	0.99			Yes		
ARHGAP32	Rho GTPase activating protein 32	4.42057896	1			Yes		Yes
ARHGAP29	Rho GTPase-activating protein 29	4.38910484	1		Apical to TJs (33)	Yes		Yes
PICALM	Phosphatidylinositol binding clathrin assembly protein	4.31826401	1					
SH3KBP1	SH3 domain containing kinase binding protein 1=CIN85=Ruk	4.26722431	1					
CRIP2	Cysteine-rich protein 2 Lim Domain containing	4.20174789	0.99			Yes		
TLN1	Talin 1	4.02423763	0.99		FAs (34)			
MYO1B	Myosin IB	3.97839737	0.99			Yes		
ABLIM2	Actin binding LIM protein family member 2	3.91814613	0.99			Yes		
HNRNPK	Heterogeneous nuclear ribonucleoprotein K	3.8900795	0.93					
XRN1	5′-3′ exoribonuclease 1	3.85476017	0.99					
LRRFIP1	Binds nucleic acids	3.63029671	1					
PTPN11	Protein-tyrosine-phosphatase=Shp-2	3.57486153	0.99		AJs (35)			
ABR	Active breakpoint cluster region-related protein	3.5374918	0.99			Yes		
FLNB	Filamin B	3.48931217	1			Yes		
TRIM25	Tripartite motif containing 25 a ubiquitin E3 ligase	3.40487194	0.9					
NEDD4L	E3 ubiquitin-protein ligase (EC 2.3.2.26)	3.33586884	1					
DIAPH2	Diaphanous related formin 2	3.00013924	0.99		TJs (36)	Yes		
VCL	Vinculin	2.9792738	1		AJs/FAs (37)			
TNKS1BP1	Tankyrase 1 binding protein 1	2.96853542	0.99					
ANK3	Ankyrin 3	2.44569302	1	Yes		Yes		
PXN	Paxillin	2.39988899	0.99		FAs (38)			
PTBP1	Polypyrimidine tract-binding protein 1	2.32428741	1					

Column A includes the official gene names of the proteins identified. Column B includes the protein name used by the community. In some cases two alternative names are included if both are widely used. Bold=known interactor. Column B highlighting. Green highlighting=protein bait. Orange highlighting=proteins with functions that seem unlikely to be associated with cell junctions. Column C. Average Log2 Fold Change in BL-Afadin sample versus BirA alone control sample. Column D. SAINT probability score. Column E. Proteins where biotinylated peptides were detected in the mass spectroscopy experiment. Column F. Proteins known to localize to a particular cell junctions, along with a literature reference found in the supplemental information. AJ=adherens junction. TJ=tight junction. FA=focal adhesion. Column G. Proteins known to associate with or regulate the cytoskeleton. Column H. Proteins overlapping with the BioID list in Goudreault et al. (2022). Column H. Proteins overlapping with the BioID list in Go et al. (2021).

**Table 2.** Validated interactors with Afadin-BL

Gene name	Protein name	AVE Log2 fold change versus control	SAINT probability	Biotinylated	Junctional protein	Cytoskeleton associated or regulating	Scribble list	Global BIO ID
Afdn	Afadin (Protein Af-6)	11.8694696	1	Yes	AJs			
TJP1	**Tight junction protein 1=ZO-1**	11.1332531	1	Yes	AJs (1)			Yes
TJP2	**Tight junction protein 2=ZO-2**	9.49723148	1		AJs (3)			Yes
NECTIN3	**Nectin cell adhesion molecule 3**	9.32149029	1	Yes	AJs (18)		Yes	
SORBS3	Sorbin and SH3 domain containing 3 (Vinexin)	9.29414558	1		FAs (39)			
CXADR	CXADR Ig-like cell adhesion molecule	9.05606031	1		AJs/TJs (40)		Yes	
ERBIN	Erbb2 interacting protein	8.5976696	1	Yes	AJs (5)			Yes
KIAA1217	KIAA1217	8.58165884	1					Yes
NECTIN2	**Nectin cell adhesion molecule 2**	8.39516926	1		Yes/AJs (27)		Yes	Yes
CTNND1	Catenin delta 1=p120	8.38013363	1		AJs (12)		Yes	Yes
SORBS2	**Sorbin and SH3 domain containing 2**	8.37665606	1	Yes	AJs/Desmosomes (2)			
ISG15	ISG15 ubiquitin like modifier	8.27373981	1					
TXNL1	Thioredoxin like 1	7.90714931	1					Yes
USP6NL	USP6 N-terminal like	7.83636522	1	Yes			Yes	Yes
LZTS2	Leucine zipper putative tumor suppressor 2 (Protein LAPSER1)	7.71385765	1	Yes			Yes	Yes
CCDC85C	Coiled-coil domain containing 85C	7.49057674	0.98		AJs (41)		Yes	Yes
ZDHHC5	Palmitoyltransferase ZDHHC5	7.41904163	1	Yes			Yes	
PARD3B	Par-3 family cell polarity regulator beta	7.315979	1		AJs/TJs (11)			Yes
MAGI3	Membrane-associated guanylate kinase, WW and PDZ domain-containing protein 3	7.29737806	1		AJs/TJs (42)			
EPS8L2	EPS8 like 2	7.24538469	1					
SCRIB	**Scribble planar cell polarity protein**	6.98193264	1	Yes	AJs (7)		Yes	Yes
RAP1A	**Small monomeric GTPase**	6.92673683	1					
GRB2	Growth factor receptor bound protein 2	6.84714794	1					
PAK4	**Serine/threonine protein kinase**	6.72623634	1	Yes	AJs (43)		Yes	Yes
SLITRK4	SLIT and NTRK like family member 4	6.52839088	1				Yes	
FNBP1L	Formin-binding protein 1-like (TOCA-1)	6.49848366	1		TJs (44)	Yes		
TP53BP2	**Tumor protein p53 binding protein 2**	6.22128868	1		AJs (9)		Yes	Yes
RRBP1	Ribosome binding protein 1	6.01814175	1					
EIF4B	Eukaryotic translation initiation factor 4B	6.00466537	1					
DBNL	Drebrin like	5.98423052	0.99	Yes		Yes		
SNAP23	Synaptosomal-associated protein	5.94034195	0.99				Yes	
SWAP70	Switching B cell complex subunit 70	5.8987627	0.93	Yes		Yes		
PPP1R13B	Protein phosphatase 1 regulatory subunit 13B	5.8618741	0.99				Yes	Yes
FRMD4B	FERM domain containing 4B	5.84554768	1					Yes
PLEKHA5	Pleckstrin homology domain containing A5	5.83812809	1				Yes	Yes
MAGI1	Membrane associated guanylate kinase, WW and PDZ domain containing 1	5.8365407	1		Ajs/TJs (45)			Yes
PLEKHA1	Pleckstrin homology domain containing A1	5.80003405	0.91					Yes
PPL	Periplakin	5.79214382	1		Desmosomes (24)			
PLEKHA6	Pleckstrin homology domain containing A6	5.71465969	1		AJs (15)			
CTTN	Cortactin	5.65959549	0.99		AJs/Desmosomes (23)	Yes		
DVL3	Dishevelled segment polarity protein 3	5.62817192	0.99		TJs (23)			
PALM	Paralemmin-1	5.50338936	1					
DPYSL2	Dihydropyrimidinase-related protein 2	5.50270367	1			Yes		
LPP	LIM domain containing preferred translocation partner in lipoma	5.49694538	0.99		AJs (8)			
CRK	Cytoplasmic adapter protein	5.42787361	1	Yes				
TJP3	**Tight junction protein 3**	5.42333508	1		Ajs/TJs (6)			
CRKL	CRK like proto-oncogene	5.42263699	1	Yes				
PRUNE1	Prune exopolyphosphatase 1	5.35961294	0.99					
MPRIP	Myosin phosphatase Rho interacting protein	5.21374273	0.98			Yes		
CD2AP	CD2 associated protein	5.1956625	1			Yes		
LUZP1	Leucine zipper protein 1	5.15895176	1			Yes		
PKP4	Plakophilin 4	5.10377026	1		Desmosomes (46)		Yes	Yes
EPB41L1	Erythrocyte membrane protein band 4.1 like 1	5.0229845	1			Yes		
LAD1	Ladinin 1	4.99046707	1			Yes		
CTNNA1	**Catenin alpha 1**	4.97055149	1		Ajs (17)		Yes	Yes
FAM91A1	Family with sequence similarity 91 member A1	4.68596554	1	Yes				
PAK2	Serine/threonine protein kinase	4.61646938	1		AJs/TJs (47)			
NCK1	Cytoplasmic adapter protein	4.54878139	1	Yes				
PPP1CB	Serine/threonine-protein phosphatase	4.54652596	1					
PFKL	ATP-dependent 6-phosphofructokinase	4.53705597	1					
DLG1	Discs large homolog 1	4.52658367	0.99		Ajs (26)		Yes	Yes
LARP1	La ribonucleoprotein 1, translational regulator	4.50182438	0.95					
PLEKHA7	**Pleckstrin homology domain containing A7**	4.49379158	1		AJs (48)			Yes
LASP1	LIM and SH3 domain protein 1	4.48109436	1		Ajs (25)			
EXOC3	Exocyst complex component 3	4.46197319	0.99					
MARK2	Serine/threonine protein kinase	4.39980412	0.99	Yes			Yes	Yes
BRD4	Bromodomain containing 4	4.15370369	0.91					
CGN	Cingulin	4.1398077	1		TJs (10)			Yes
UACA	Uveal autoantigen with coiled-coil domains and ankyrin repeats	4.13255405	1					Yes
SF1	Splicing factor 1	4.08001041	0.99					
ABLIM3	Actin binding LIM protein family member 3	4.07724667	0.99	Yes	Ajs (13)	Yes		
FERMT2	FERM domain containing kindlin 2=Mig2	4.04913902	0.99		FAs (49)			
SIPA1L1	Signal induced proliferation associated 1 like 1	3.98044682	1					Yes
VASP	Vasodilator stimulated phosphoprotein	3.96868229	1			Yes		
ANK3	Ankyrin 3	3.96721458	1	Yes		Yes		
KHDRBS1	KH RNA binding domain containing, signal transduction associated 1	3.90842533	0.91					
RSU1	Ras suppressor protein 1	3.88080311	0.99		FAs (50)			
VCPIP1	Ubiquitinyl hydrolase 1	3.86772728	0.95	Yes				
EPB41	Erythrocyte membrane protein band 4.1	3.79453564	1		TJs (31)	Yes		
ARHGAP29	Rho GTPase-activating protein 29	3.61454391	0.99		Apical to TJs (33)	Yes		Yes
GAB1	GRB2 associated binding protein 1	3.61007404	0.98					Yes
DNAJB1	DnaJ heat shock protein family (Hsp40) member B1	3.60828114	0.98					
ANXA1	Annexin	3.6010437	0.96	Yes				
STOM	Stomatin	3.45711517	0.99					
RASSF8	Ras association domain family member 8	3.35538387	0.99		AJs (32)		Yes	Yes
SEPTIN9	Septin 9	3.34074402	0.98					
PICALM	Phosphatidylinositol binding clathrin assembly protein	3.2706604	0.99					
CSNK1A1	serine/threonine protein kinase	3.19583607	0.98					
PARD3	Par-3 family cell polarity regulator	3.17638779	1		AJs/TJs (51)			Yes
NIBAN2	Niban apoptosis regulator 2=FAM129B	3.15076065	0.99		AJs (52)			
SH3KBP1	SH3 domain containing kinase binding protein 1	3.07292461	0.98					
ERC1	ELKS/RAB6-interacting/CAST family member 1	3.07198429	1					
FXR1	FMR1 autosomal homolog 1	2.98178196	0.98					
ARHGAP32	Rho GTPase activating protein 32	2.8637619	0.99			Yes		Yes
TWF2	Twinfilin actin binding protein 2	2.84425354	0.99			Yes		
TRIM25	Tripartite motif containing 25	2.71160793	0.95					Yes
PPP2R2A	Serine/threonine-protein phosphatase 2A 55 kDa regulatory subunit B	2.69836235	1		Desmosomes (53)			
DHX9	RNA helicase DEAH box protein 9	2.65321445	0.98					
CARMIL1	Capping protein regulator and myosin 1 linker 1	2.46775579	0.99			Yes		Yes
PTBP1	Polypyrimidine tract-binding protein 1	2.34704685	0.99					
DDX17	DEAD-box helicase 17	2.34253979	0.99					
RAB5B	Small monomeric GTPase	2.2430582	0.98					
PTPN11	Protein-tyrosine-phosphatase=Shp-2	2.02278805	1		AJs (35)			
PGK1	Phosphoglycerate kinase	1.99775982	0.99				Yes	
KIF5B	Kinesin-like protein	1.914258	0.99					
CDK6	Cyclin dependent kinase 6	1.7561903	0.98					
ACSS1	Acetyl-coenzyme A synthetase	1.69136429	1					

Column A includes the official gene names of the proteins identified. Column B includes the protein name used by the community. In some cases two alternative names are included if both are widely used. Bold=known interactor. Column B highlighting. Green highlighting=protein bait. Orange highlighting=proteins with functions that seem unlikely to be associated with cell junctions. Column C. Average Log2 fold change in Afadin-BL sample versus BirA alone control sample. Column D. SAINT probability score. Column E. Proteins where biotinylated peptides were detected in the mass spectroscopy experiment. Column F. Proteins known to localize to a particular cell junctions, along with a literature reference found in the supplemental information. AJ=adherens junction. TJ=tight junction. FA=focal adhesion. Column G. Proteins known to associate with or regulate the cytoskeleton. Column H. Proteins overlapping with the BioID list in Goudreault et al. (2022). Column H. Proteins overlapping with the BioID list in Go et al. (2021).

**Table 3.** Overlap between the BL-Afadin and Afadin-BL lists (arranged alphabetically)

BL-Afadin full list	Afadin-BL full list	On both lists
ABLIM1	ABLIM3	ABLIM3
ABLIM2	ACSS1	ANK3
ABLIM3	ANK3	ARHGAP29
ABR	ANXA1	ARHGAP32
ANK3	ARHGAP29	CD2AP
ARHGAP29	ARHGAP32	CGN
ARHGAP32	BRD4	CTNNA1
CD2AP	CARMIL1	CTNND1
CGN	CCDC85C	CTTN
CLMN	CD2AP	DBNL
COBLL1	CDK6	DLG1
CORO1C	CGN	DPYSL2
CRIP2	CRK	DVL3
CRK	CRKL	EPB41
CRKL	CSNK1A1	EPB41L1
CTNNA1	CTNNA1	EPS8L2
CTNND1	CTNND1	ERBIN
CTTN	CTTN	FAM91A1
DBN1	CXADR	FRMD4B
DBNL	DBNL	ISG15
DIAPH2	DDX17	KIAA1217
DLG1	DHX9	LAD1
DPYSL2	DLG1	LASP1
DVL3	DNAJB1	LPP
EIF4ENIF1	DPYSL2	LUZP1
EPB41	DVL3	LZTS2
EPB41L1	EIF4B	NCK1
EPS8L2	EPB41	NECTIN2
ERBIN	EPB41L1	NECTIN3
FAM91A1	EPS8L2	PARD3B
FLNB	ERBIN	PFKL
FRMD4B	ERC1	PICALM
HNRNPK	EXOC3	PLEKHA5
ISG15	FAM91A1	PLEKHA6
KANK2	FERMT2	PPL
KIAA1217	FNBP1L	PPP1CB
KIAA1522	FRMD4B	PTBP1
KIAA1671	FXR1	PTPN11
LAD1	GAB1	RAP1A
LASP1	GRB2	RASSF8
LIMA1	ISG15	SCRIB
LMO7	KHDRBS1	SEPTIN9
LPP	KIAA1217	SF1
LRRFIP1	KIF5B	SH3KBP1
LUZP1	LAD1	SIPA1L1
LZTS2 LAPSER1	LARP1	SORBS2
MYO1B	LASP1	SWAP70
NCK1	LPP	TJP1
NECTIN2	LUZP1	TJP2
NECTIN3	LZTS2	TJP3
NEDD4L	MAGI1	TP53BP2
OAS1	MAGI3	TRIM25
PARD3B	MARK2	USP6NL
PDLIM5	MPRIP	VASP
PFKL	NCK1	VCPIP1
PICALM	NECTIN2	
PLEKHA5	NECTIN3	
PLEKHA6	NIBAN2	
PPL	PAK2	
PPP1CB	PAK4	
PTBP1	PALM	
PTPN11	PARD3	
PXN	PARD3B	
RAP1A	PFKL	
RASSF8	PGK1	
SCRIB	PICALM	
SEPTIN9	PKP4	
SF1	PLEKHA1	
SH3KBP1	PLEKHA5	
SH3RF1	PLEKHA6	
SHROOM2	PLEKHA7	
SHROOM3	PPL	
SIPA1L1	PPP1CB	
SIRT2	PPP1R13B	
SLC25A1	PPP2R2A	
SORBS1	PRUNE1	
SORBS2	PTBP1	
STAT3	PTPN11	
SWAP70	RAB5B	
TJP1	RAP1A	
TJP2	RASSF8	
TJP3	RRBP1	
TLN1	RSU1	
TNKS1BP1	SCRIB	
TP53BP2	SEPTIN9	
TRIM25	SF1	
USP6NL	SH3KBP1	
USP7	SIPA1L1	
UTRN	SLITRK4	
VASP	SNAP23	
VCL	SORBS2	
VCPIP1	SORBS3	
WDR11	STOM	
XRN1	SWAP70	
ZYX	TJP1	
	TJP2	
	TJP3	
	TP53BP2	
	TRIM25	
	TWF2	
	TXNL1	
	UACA	
	USP6NL	
	VASP	
	VCPIP1	
	ZDHHC5	

### Our lists contain many known interactors and are highly enriched for known junctional and cytoskeletal proteins

As a first verification of the quality of our data, we asked whether the lists included proteins known to interact with Afadin directly or via co-immunoprecipitation. The results were striking. Nectins were the first proteins found to interact with Afadin (Takahashi et al., 1999) and both Nectin2 and Nectin3 were on our lists, with SAINT probabilities of 1, 1 or 0.99, 1 for N- or C terminal tagged Afadin, respectively. The known binding partner Alpha-catenin (CTNNA1; Pokutta et al., 2002) was also on both N- and C-terminal lists, as was another component of the cadherin-catenin complex, p120 (CTNND1). All three ZO-1 family members [TJP1 (Yamamoto et al., 1997), TJP2, TJP3] were on both lists, as was Rap1A, which binds to Afadin's N-terminal RA domain (Wohlgemuth et al., 2005) and activates Afadin. Two known interactors identified in previous BioID screens, Scribble (Goudreault et al., 2022) and Pak4 (Baskaran et al., 2021), were also on our lists. Pak4 was only identified with our C-terminally tagged Afadin. Four other known interactors were also included: TP53BP2/ASPP2 (Royer et al., 2022), identified with both baits, LMO7 (Ooshio et al., 2004), SORBS1/Ponsin (Mandai et al., 1999), all scoring positive only with the N-terminal bait, and PLEKHA7 (Kurita et al., 2013), scoring positive only with the C-terminal bait.

**Fig. 4.**
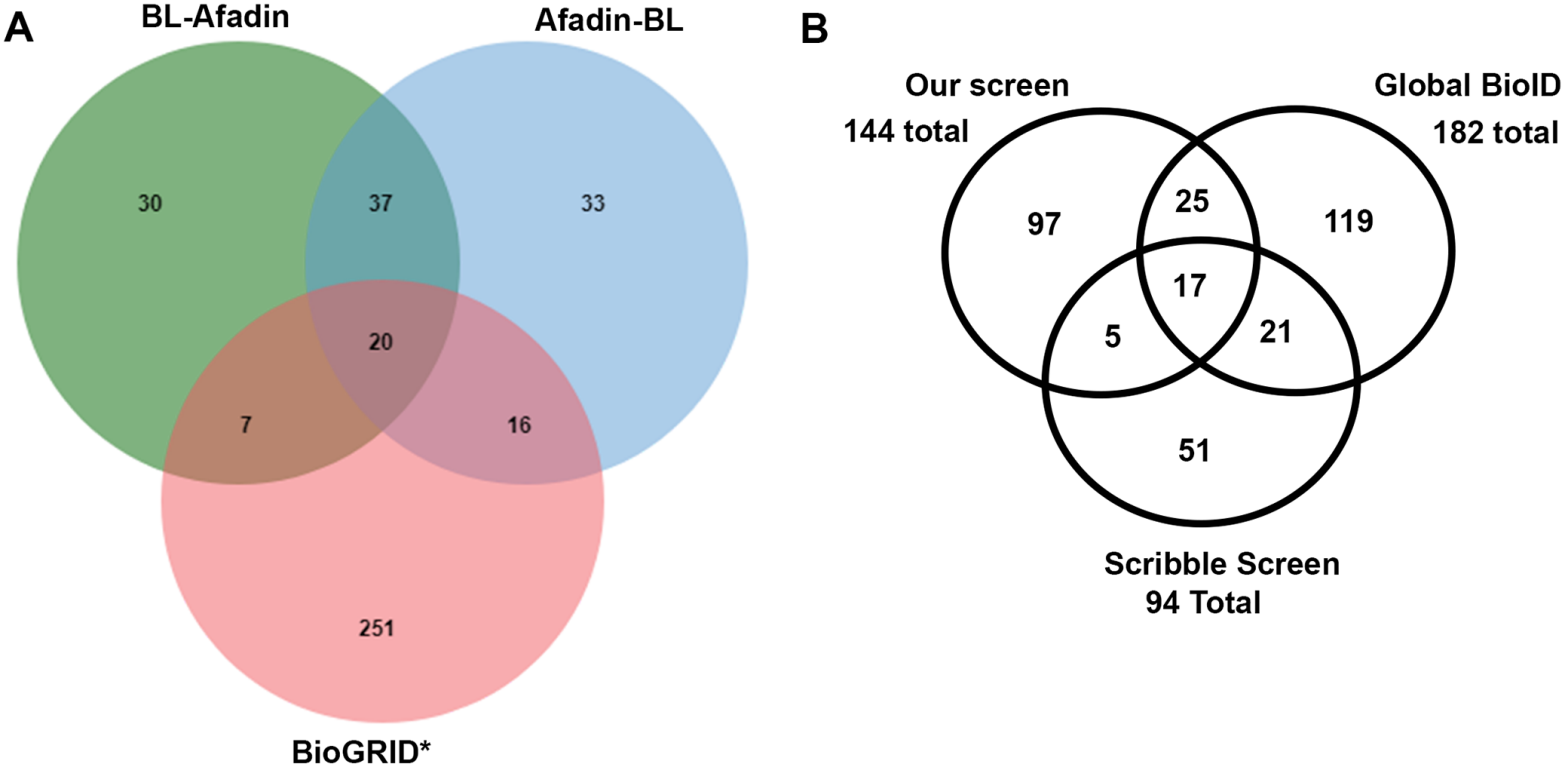
**Venn diagrams illustrating the overlap between different screens.** (A) Overlap between the full list of proteins in the BioGRID database as interacting with Afadin, and the lists of proteins identified as Afadin neighbors using BL-Afadin or Afadin-BL. (B) Overlap of the proteins identified in our screen with both baits and those identified in two earlier BioID screens with N-terminally tagged Afadin.

As a more complete assessment of the overlap between our lists and known interactors, we used The Biological General Repository for Interaction Datasets (BioGRID; Oughtred et al., 2021), a public database that archives and disseminates genetic and protein interaction data from model organisms and humans. These are curated from both high-throughput datasets and individual focused studies in the literature. Using the data from humans (the closest match to the canine cells we used), 294 proteins are annotated as interacting with Afadin genetically or via protein-protein interactions (Table S2). Of the 141 proximal proteins identified on one or both of our lists, 43 were previously reported as physically co-complexed with Afadin within the BioGRID database (Oughtred et al., 2021). These include seven found only on our BL-Afadin list, 16 found only on our Afadin-BL list, and 20 proteins found on both (Fig. 4A; Table S2).

Next, we used the Panther 19.0 classification system (Thomas et al., 2022) to determine the Gene Ontology (GO) biological processes over-represented in our lists relative to the full set of 20,580 proteins in the human genome. We used proteins with a SAINT score=1 from the BL-Afadin or Afadin-BL lists. Each list was enriched for similar but not identical biological processes. The most enriched process on the BL-Afadin list was ‘cytoskeletal organization’, followed by ‘actin-filament-based process’ and ‘actin cytoskeleton organization’ (Table S3). ‘Cell adhesion’ was the fifth most enriched process, and ‘cell junction organization’ and ‘cell-cell adhesion’ were in the top ten, along with two processes involved in ‘barrier’ function. The most enriched process on the Afadin-BL list was ‘cell-cell junction organization’, with ‘cell junction organization’, ‘cell adhesion’ and ‘cell-cell adhesion’, and ‘protein localization to cell-cell junction’ ranked third, fourth, fifth, and sixth, while two processes involved in ‘barrier’ function were in the top ten (Table S4). ‘Cytoskeletal organization’ was ranked second. Thus, our lists are statistically strongly enriched for cell junction and cytoskeletal proteins.

We also manually examined the literature to examine what was known about the subcellular localization of the hits with SAINT scores ≥0.9 on the BL-Afadin, Afadin-BL, or both lists (Tables 1 and 2; total=146 proteins on one, the other, or both lists). 39 proteins are reported to localize to AJs, and an additional 14 are known to localize to tight junctions (some are reported to localize to both). Intriguingly, our lists also included five proteins reported to localize to desmosomes, including two well-known desmosomal adapter proteins, Periplakin and Plakophilin4. The lists also include many proteins known to regulate or associate with the actomyosin cytoskeleton – 39 proteins fit that category.

### Afadin proximal proteins enriched in ZOdKD MDCK cells

One of the secondary aims of our experiments was to compare the protein neighbors of Afadin in wild-type MDCK cells and in MDCK cells depleted for the tight junction proteins of the ZO-1 family (ZO-KD cells). In our previous work we found that shRNAi-based knockdown of ZO-1 and ZO-2 dramatically altered junctional architecture (Choi et al., 2016; Fanning et al., 2012). Cell junctions became much straighter, and robust sarcomeric arrays of actin and myosin assembled along AJs. In response to the elevated junctional tension, Afadin recruitment to junctions was elevated and it was important for maintaining epithelial architecture in these cells. We thus wondered if the protein neighbors of Afadin would be altered, so we compared differential enrichment of proteins in wild-type MDCK cells versus ZO-KD cells. These data are presented in Table S1 on the ‘Differentially Proximal’ tab and can be sorted by degree of enrichment in the comparison of the two cell lines for both N-terminally tagged and C-terminally tagged Afadin. One limitation of this analysis was that we only had a single replicate of the ZO-KD samples, and thus combined the BL-Afadin and Afadin-BL samples in this comparison. This also meant fewer proteins met the SAINT ≥0.9 threshold (Table S1, Proximal Proteins tab). Another issue is that expression of tagged Afadin constructs did appear to elevate junctional F-actin (Fig. 2). However, this level of Afadin overexpression did not lead to the straightened cell boundaries seen after ZOdKD, either in our previous work using a GFP-tagged Afadin (Choi et al., 2016) or in the work described here. We thus do not think we are seeing the same level of elevated junctional contractility but cannot rule out that this made our parental MDCK cells more similar to our ZOdKD cells.

In examining differential expression, we hoped to find proteins that work together to strengthen cell-cell junctions in response to elevated tension. However, we were surprised to find relatively few differences. As expected, we observed lower enrichment of ZO-1 and ZO-2 (also known as TJP1 and TJP2), the two proteins targeted by siRNA. These proteins were decreased by 5.3- and 7.6-fold, respectively. ZO-3 was also less enriched, consistent with the fact that ZO-3 is less stable when the other family members are knocked down (Fanning et al., 2012). However, the list of proteins relatively enriched in ZO-KD cells was not as informative as we had anticipated. 30 of the 41 proteins with a SAINT score ≥0.9 identified in our experiments with ZOdKD cells were shared with either the BL-Afadin or Afadin-BL lists from the parental MDCK cells. Combining the differential lists for our N- and C-terminally tagged Afadin constructs, the most enriched protein in ZOdKD cells was a protein about which very little is known (ERICH6B). Other highly ranked differentially accumulating proteins include enzymes with no clear connection to junctions, including acetyl-CoA carboxylase (ACACB), a component of the pyruvate dehydrogenase complex (DBT), the mitochondrial acyl-CoA ligase ACSF2, or a cytochrome p450 enzyme (CYP4A37), as well as the endoplasmic-reticulum localized Inositol 1,4,5-trisphosphate receptor (ITPR3). Further, this ‘differential enrichment’ reflects in part the fact that none of these six proteins reached a SAINT score of ≥0.9 in our parental MDCK cells, and five of the six had SAINT scores lower than 1.0 in our ZOdKD cells – thus we think they may be false positives,

However, the differentially enriched list did provide some candidates to explore to define how ZOdKD alters junctional architecture. Shroom3, which is recruited to cell junctions in ZOdKD cells and which is important for the elevated junctional contractility and thus straightened cell junctions in those cells (Choi et al., 2016), was enriched in the ZOdKD cells (1.8-fold), as was its paralog Shroom2 (1.9-fold), consistent with our previous observations. One interesting upregulated protein was Vinculin, which is known to be recruited to AJs under elevated tension and which we found enriched at AJs after ZO-KD (Choi et al., 2016). However, in our current dataset this did not get a SAINT score of ≥0.9. There were, however, a set of other upregulated proteins that will be important to investigate in the future as candidate junctional stabilizers. These include the junctional Armadillo-family protein Plakophilin 4 (5.0-fold enriched in our N-terminally tagged set and 3.6-fold enriched in our C-terminally tagged set) Rho-family GAP ARHGAP32 (3.6- and 2.9-fold enriched, respectively), SORBS2 (2.3- and 2.5-fold enriched, respectively), and LIM domain and actin binding protein 1 (LIMA1; 2.8- and 2.0-fold enriched). In addition, the known interactor Pak4 was also enriched after ZOdKD (1.9- and 1.6-fold), as was Erbin (2.6- and 1.7-fold), a paralog of the known interactor Scribble.

The results were similar when we examined proteins differentially enriched in wild-type MDCK cells, and thus downregulated after ZOdKD. Two of the most upregulated proteins seemed to have little connection to cell junctions or the cytoskeleton, like the ISG15 ubiquitin like protein ISG15 and the translational regulator LARP1. However, others were more intriguing, including Zyxin (up 4.2-fold) and VASP (up 3.5-fold), which are both recruited to actin filaments after stress or damage (Smith et al., 2010), or MAGI3 (up 4.0-fold), the homolog of which regulates AJs and cell shape changes in the *Drosophila* eye (Zaessinger et al., 2015). Following up on these differences may help explain the dramatic differences in junctional architecture in these two cell lines.

### Comparing our screen to previous proximity labeling screens

Two previous papers reported the results of proximity labeling screens using Afadin as a bait. Both used a single bait, with Afadin N-terminally tagged. We compared our results to theirs. The first screen was part of a large-scale proteomics effort in which more than 200 intracellular proteins from 32 different cellular compartments were tagged with BirA* and expressed in HEK293 cells (Go et al., 2021). N-terminally tagged Afadin was one of their baits. 26 of the 95 hits from our screen with BL-Afadin with a SAINT score ≥0.9 were also on their list (Table 1) and 34 of the 106 hits from our screen with Afadin-BL with a SAINT score ≥0.9 from our screen were also on their list (Table 2), for a total of 42 shared proteins (Fig. 4B; Table S6), revealing strong overlap. This direct comparison likely understates the overlap, as in many cases one list includes paralogs of proteins on the other list, e.g. Nectin 2 versus Nectin 3 or SorbS1 versus SorbS2. These would add 13 more matches. A second group was interested in Afadin's role as a Ras GTPase effector and carried out a proximity proteomics screen using N-terminally tagged Afadin as a bait in HeLa cells (Goudreault et al., 2022). Here overlap was present but less pronounced. Only 12 of 95 hits from our screen with BL-Afadin with a SAINT score ≥0.9 (Table 1), and 21 of 106 hits from our screen with Afadin-BL with a SAINT score ≥0.9 (Table 2) were also on their list, for a total of 22 shared proteins (Fig. 4B; Table S6).

Intriguingly, each screen lacked some proteins seen in other screens. Only 17 proteins were found in all three screens (Table S6), and this list only included five of the 14 known interactors identified in our screen, lacking for example ZO-1 family proteins. Among the proteins shared by our list and the Global list, there were 43 proteins shared by both of these screens, and the shared set now included eight of 14 known interactors identified on our lists. As we discuss below, the three screens used three different cell types, likely explaining some of these differences.

## DISCUSSION

Afadin and its *Drosophila* homolog Canoe are key components of the protein network that links AJs to the actomyosin cytoskeleton. They stabilize this connection as force is generated, thus helping ensure cell shape change and movement during embryonic morphogenesis, organogenesis and tissue homeostasis, without tissue disruption. While initial work suggested E-cadherin links to actin via a simple direct connection involving beta- and alpha-catenin, we now know this connection involves many more proteins linked by multivalent interactions. Many proteins in this network have been defined, but others are likely to be involved. Proximity proteomics provides a powerful tool to identify potential new players.

### Multiple proximity proteomics screens identify overlapping but not identical proteins

Our screen was the third to use N-terminally tagged Afadin as a bait. Several criteria suggest our screen was of high quality. First, we identified 14 known interacting proteins, including the many known physical interactors identified by direct protein interactions or co-immunoprecipitation, along with 43 proteins on the BioGRID interactor list (Table S2). It is possible some known interactors that were not identified, like Nectin1, are not expressed at high enough levels in MDCK cells. Second, when we used the Panther 19.0 classification system to assess the biological processes in which our identified proteins participated, the top categories almost all reflected cell junctions or the cytoskeleton (Tables S3 and S4).

Each of the three screens identified overlapping sets of proteins, with each also including some unique potential protein neighbors. The overlap with one of the previous screens was particularly strong, with 42 out of 146 proteins on at least one of our lists (BL-Afadin or Afadin-BL SAINT≥0.9) shared with that screen (Fig. 4B; Table S6). However, list of proteins overlapping in all three screens (17) or in the two most extensive screens (42), and the fact that neither of these overlapping lists included all known interactors found in our screen, suggests the combined lists are a more useful resource than any single list alone. Another value of the multiple screens is that they targeted different cell types [MDCK cells (our screen), HeLa cells (Goudreault et al., 2022), or HEK293 cells (Go et al., 2021)]. This helps identify tissue- or cell type-specific interactors. It is worth noting that HeLa cells, a cervical cancer cell line, do not form fully functional tight junctions and therefore lack robust epithelial barrier function (Shi et al., 2020). Likewise, HEK293 cells do not form tight junctions and are often used as a ‘tight junction free’ background in which to test the function of tight junction proteins like claudins (Inai et al., 2009). The absence of tight junctions seems likely to alter the molecular architecture of other cell junctions. Other proteomics approaches also will provide insights. Afadin was one of the proteins analyzed in a large scale IP-mass spectroscopy screen (Huttlin et al., 2021) – of the 22 proteins identified by IP-mass spectroscopy, eight were shared with our lists (Table S6), but this screen also identified additional proteins we did not identify, and only one of the proteins common between our screen and the IP-mass spectroscopy screen, CXADR, was found in the overlap between all three different proximity proteomics screens.

### How did using both N-terminal and C-terminal baits inform our understanding?

The use of bait proteins tagged with BirA* in different positions has the theoretical advantage of identifying proteins interacting with different protein domains positioned along the N- to C-terminal axis, or that are differentially positioned in a multiprotein complex. Afadin is a relatively large protein (1882 amino acids) with five folded protein domains and a long C-terminal intrinsically disordered region (IDR), and thus the potential distance between an N- and C-terminal BirA* tag is substantial. The 800 aa IDR alone could reach ∼280 nm if fully extended and the theoretical labeling radius of BirA* is ∼10-50 nm (Bosch et al., 2021). However, we did not see dramatic differences between the proteins identified with our two baits. 58 of 145 proteins with SAINT scores ≥0.9 were present on both lists. 38 only passed that SAINT score threshold on the BL-Afadin list, and 49 only passed that SAINT score threshold on the Afadin-BL list. We also analyzed overall differential enrichment of all of the proteins we identified (Table S1, ‘Differential enrichment’). These differentially enriched proteins may provide clues as to positioning relative to different domains or regions of Afadin – for example, both Nectins we identified, which interact with the PDZ domain, were only found in the Afadin-BL sample, consistent with the idea that the PDZ is the most C-terminal of the folded domains and the IDR may be very flexible. It will be interesting to determine the nature of the interaction between Pak4 and Afadin, as it was the protein most differentially enriched in the Afadin-BL sample (6-fold; Table S1, ‘Differential enrichment’). In contrast, three of the LIM domain proteins in the dataset, LMO7, Zyxin, and PDLIM7, were all differentially enriched in the BL-Afadin sample (3.8- to 4.8-fold; Table S1, ‘Differential enrichment’). Whether they interact directly or indirectly, and if directly, their sites of interaction with Afadin, all remain unknown. At least one differentially-enriched protein, ZO-3, was puzzling given what we know. ZO-1 family proteins can interact with a proline-rich region in the IDR (Ooshio et al., 2010), but ZO-3 was differentially enriched in the BL-Afadin sample (3.5-fold; Table S1, ‘Differential enrichment’), which does not fit with this binding site. However, neither of the other ZO-1 family proteins met the threshold of 2-fold differential enrichment.

Several things may explain the strong overlap of proteins identified with the two baits. First, if the IDR is as flexible and extendable as predicted, the C-terminal BirA* may be able to reach proteins throughout the neighborhood. Second, it has become increasingly clear that connections between the cadherin tail and actin do not occur through simple linear connections, as once thought, but involve a large network of proteins linked by multivalent connections. Further, many of the proteins in the linkage network can phase separate (Sun et al., 2022), including Afadin (Kuno et al., 2024 preprint), and junctional puncta contain hundreds to thousands of junctional proteins (McGill et al., 2009). Within these potential biomolecular condensates, each Afadin may be in a somewhat different environment, with different binding partners and different neighbors. This contrasts with a model in which each individual Afadin is bound to all of its known binding partners simultaneously, with these fixed interactions creating a more ‘crystalline’ interaction network. In fact, when examining a protein with a long IDR, different parts of the same protein may localize differently – this was observed when using super-resolution microscopy to localize N- versus C-terminally tagged ZO-1 (Nguyen et al., 2024; Spadaro et al., 2017).

### Proteomics suggest proteins from ‘different cell junctions’ can be juxtaposed

Textbooks depict the lateral borders of epithelial cells as lined by discrete sets of cell junctions. Apical tight junctions, their more basal neighbors the AJs and the desmosomes found in some epithelial cells, were initially defined by electron microscopy, with cellular and molecular studies adding lists of core components and associated proteins. Recent work added more nuance to this, with high resolution microscopy placing the Crumbs complex even more apical in the ‘marginal zone’ (Martin et al., 2021) and most recently the separation of the AJ into zones enriched for Afadin/Nectins and the cadherin-catenin complex (Mangeol et al., 2024).

Proximity proteomics provides a tool to explore the distinctions between these junctions and offered some surprises. Van Itallie et al. used proximity proteomics to explore proteins in the vicinity of the core AJ protein E-cadherin (Van Itallie et al., 2014) or in the vicinity of the protein often used as the tight junction marker, ZO-1 (Van Itallie et al., 2013). When we examined the top 125 proteins in each screen, we were surprised to find more matches in the ZO-1 dataset (37 matches; Afadin ranked 15th) than in the E-cadherin dataset (29 matches; Afadin ranked 17th). Tan et al. used proximity proteomics to explore neighbors of polarity regulators in the Par complex – here they used Par3, usually viewed as a tight junction protein, as bait – or of the Crumbs complex, which defines the marginal zone, using Pals as bait (Tan et al., 2020). Consistent with the overlap of our list with that for ZO-1, 18 of 87 proteins on the Par3 list overlapped our lists, including Afadin itself, Nectin2, and alpha-catenin, all usually viewed as AJ proteins. While the Pals list did not include Afadin, it did include Nectin2, and 11 of 110 proteins on this list overlapped with ours, including the tight junction proteins ZO-1, ZO-3, Cingulin.

This suggests additional complexity. First, at the boundaries between different junctions, proteins will likely be close to proteins in the next junction more apical or basal. Recent lovely work from the Honigman lab revealed that interactions between the tight junction protein ZO-1 and the marginal zone protein Patj are key for positioning the tight junction belt apically (Pombo-García et al., 2024). It will be interesting to see if similar mechanisms underlie the recent observation that in at least some cell types Afadin and the Nectins segregate apically to the cadherin-catenin complex (Mangeol et al., 2024). Second, the neighbors of a protein may change as junctions form and mature in a single cell type – proximity proteomics of ZO-1 supports this idea (Pombo-García et al., 2024). Third, many proteins may localize to more than one junctional type, with the difference being one of degree of enrichment. This may help explain the inclusion on our list of proteins thought to be solely components of desmosomes or integrin-based focal adhesions. These include both proteins known to have a dual localization to different junctions like Plakophilin4 (desmosomes and AJs) or vinculin (focal adhesions or AJs), and proteins usually thought to be only found in desmosomes, like Periplakin, or only in integrin-based junctions, like Talin.

### Proximity proteomics opens new questions and research directions

Proximity proteomics experiments identify potential new partners of the protein of interest and offer leads for new research. The Afadin screens offer three examples. Two groups, including ours, used identification of the polarity regulator Scribble as an Afadin interactor to explore new roles for both proteins: Scribble as a key player in localizing apical AJs during *Drosophila* development (Bonello et al., 2019) and Afadin as a coupler of Ras GTPases to Scribble to regulate cell polarity and migration (Goudreault et al., 2022). Others used the Global BioID Afadin list to identify the kinase Pak4 as a regulator of junctional protein phosphorylation (Baskaran et al., 2021). Similarly, the ZO-1 BioID screen stimulated studies revealing that the BAR-domain protein TOCA-1 regulates actin assembly at tight junctions (Van Itallie et al., 2015). The new lists offer many exiting new leads to follow. Even if we confine our interests to genes shared in all three Afadin screens, there are intriguing leads. For example, the Rab5-GAP US6NL/RN-Tre regulates integrin endocytosis and focal contact turnover in mammalian cells (Palamidessi et al., 2013) and regulates assembly of myosin into contractile networks in *Drosophila* cells (Platenkamp et al., 2020) – exploring its role in cell-cell AJs seems well worth the effort. The orthologs of RASSF8 and ASPP1/ PPP1R13B form a complex regulating cell-cell adhesion during *Drosophila* retinal morphogenesis (Langton et al., 2009), working together with MAGI (Zaessinger et al., 2015), another hit in two of the screens. Looking more broadly at their roles in junctional remodeling during embryonic morphogenesis seems warranted. Little is known about the normal physiological roles of LZTS2 in either mammals or *Drosophila* – this might also be a fruitful area to pursue. We hope this dataset will stimulate further research into the roles of many of the identified proteins in cell-cell junctions in both mammals and *Drosophila*.

## MATERIALS AND METHODS

### Stable expression of BirA*-Afadin in MDCK cells

We utilized fusion proteins we had previously generated (Bonello et al., 2019). Rat Afadin coding sequences were cloned into an inducible mammalian expression vector for BioID, pTRE2hyg-BirA*-myc (Van Itallie et al., 2013). We created versions with Afadin coding sequences tagged with BirA* at the N- (pTRE2hyg-BirA*-Afadin-myc) or C-terminus (pTRE2hyg-Afadin-BirA*-myc). We then transfected subconfluent MDCK T23 cells with these plasmids, or a plasmid encoding BirA*-myc alone, and selected for the stable clones using hygromycin as a selective drug. Stable clones were verified by immunoblotting and by and immunostaining for Afadin and the Myc tag carried on the fusion proteins. We cultured cells expressing our BirA* fusions in DMEM media containing 1 g/l glucose, 10% fetal bovine serum, 15 mM HEPES (pH 7.4) and 50 ng/ml doxycycline, to keep transgene expression off.

### Immunostaining (cell culture)

MDCK and ZOdKD (Choi et al., 2016) cells expressing BioID constructs were cultured for 7 days in Transwells without doxycycline to express BirA*-Afadin fusions and fixed in ice-cold ethanol for 1 h at −20°C. After three washes with PBS, the samples were incubated with blocking buffer (10% FBS in PBS) for 1 h at room temperature. Subsequently, the inserts were incubated with primary antibodies diluted in blocking buffer. Following three washes with wash buffer (1% FBS in PBS), the cells were incubated with the Alexa-conjugated secondary antibodies along with Hoechst 33343 to stain DNA. After three washes with wash buffer, the insert membrane was cropped out, mounted on a microscope slide with Prolong Diamond antifade mountant (Thermo Fisher Scientific) and cured before imaging. The antibodies and concentrations used for immunocytochemistry (ICC) and western blotting (WB) are as follows. Primary antibodies include mouse anti-myc (9E10; 1:100 ICC, 1:100 WB) from the Developmental Studies Hybridoma Bank (DSHB); mouse anti-ZO-1 (1:100 ICC, 1:300 WB), rabbit anti-ZO-2 (1:500 WB), rabbit anti-αE catenin (1:500 WB) all from Invitrogen; rat anti-ZO-1 clone R40.76 [1:25 ICC, 1:100 WB; (Adnerson et al., 1988)]; rabbit anti-myosin IIA (1000 WB) from Covance Research Products; rabbit anti-βcatenin (1:2000 WB); rat anti-Ecad (1:500 ICC), mouse anti-AF6/afadin (1:100 ICC, 1:1,000 WB), mouse anti-α-tubulin (T6199; 1:5000 WB), phalloidin-TRITC (1:1000 ICC, F-actin probe) all from Millipore Sigma. Secondary antibodies include IR700-conjugated streptavidin (1:100 WB), IRDye 680RD and 800CW-conjugated secondary antibodies (1:10,000) from LiCor; Alexa 594-conjugated streptavidin (1:100 ICC), Alexa 488, 568, 647-conjugated secondary antibodies (1:500 ICC) from Life Technologies.

### Expression and purification of biotinylated proteins

MDCK cells were those used by Choi et al. (2016) and were authenticated and tested for contamination. MDCK cells stably expressing BirA* transgenes were cultured without doxycycline for 5 days, following which cells were treated with 50 µM biotin for 24 h. To prepare lysates, cells were washed three times with ice-cold PBS and scraped into lysis buffer [1% NP-40, 0.5% deoxycholate, 0.2% SDS, 50 mM Tris (pH 8), 150 mM NaCl, 2 mM EDTA, supplemented with protease/phosphatase inhibitor]. Lysates were snap frozen on dry ice prior to storing at −80°C. To capture biotinylated proteins, lysates were thawed at 4°C, sonicated (amplitude 50%, ten strokes performed manually) and incubated on ice for 15 min. Lysates were then spun at 15,000 ***g*** for 15 min and the protein concentration of supernatant determined using Bio-Rad Protein Assay Dye. Equal concentrations of sample were added to pre-washed Dynabeads (MyOne Streptavidin C1) and incubated with nutation overnight at 4°C. After removing the unbound sample, beads were washed twice with buffer 1 (2% SDS) for 10 min, once with buffer 2 [0.1% deoxycholate, 1% Triton X-100, 500 mM NaCl, 1 mM EDTA and 50 mM Hepes (pH 8)] for 10 min, once with buffer 3 [0.5% deoxycholate, 0.5% NP-40, 250 mM NaCl, 1 mM EDTA and 10 mM Tris (pH 8)] for 10 min and twice with buffer 4 [50 mM NaCl and 50 mM Tris (pH 7.4)] for 10 min. After last wash, the beads were incubated with 5 mM DTT for 15 min at 60°C. The beads slurry was applied to a spin filter column (VIVACON 500, 30,000 MWCO, Thermo Fisher Scientific) and centrifuged at 10,000× ***g*** for 20 min. The column was washed three times with 8 M urea, and incubated in 100 μl of chloroacetamide:urea (1:9) solution for 20 min in the dark, followed by rinse twice with 8 M urea. The denatured proteins were stabilized by washing twice with 50 mM ammonium bicarbonate and subjected to trypsin digestion (Promega, V115C) by incubating at 37°C overnight. The tryptic peptides were collected by centrifugation at 10,000× ***g*** for 10 min, and the bead-trapped peptides were eluted with a high-temperature, high-organic method [0.5% trifluoroacetic acid/acetonitrile (4:6) at 65°C for 30 min]. The collected peptides were cleaned with C-18 spin column, vacuum dried and reconstituted in Buffer A (0.1% TFA in ddH2O). The dissolved peptides were loaded onto the mass spectrometer.

### Mass spectrometry data acquisition

Trypsinized peptides were separated via reverse-phase nano-HPLC using a nanoAquity ultra performance liquid chromatography (UPLC) system (Waters Corp.). Peptides were first trapped in a 2-cm trapping column (Acclaim^®^ PepMap 100, C18 beads of 3.0-μm particle size, 100-Å pore size) and a 25-cm EASY-spray analytical column (75-μm inner diameter, C18 beads of 2.0-μm particle size, 100-Å pore size) at 35°C. The flow rate was 250 nl/min over a gradient of 5% buffer B (0.1% formic acid in acetonitrile) to 35% buffer B in 150 min, and an in Orbitrap Elite mass spectrometer (Thermo Fisher Scientific) performed mass spectral analysis. The ion source was operated at 2.4-2.8 kV with the ion transfer tube temperature set at 300°C. MS1 spectra (300-2000 m/*z*) were acquired by the Orbitrap analyzer with 120,000 resolution. MS2 spectra were acquired in data-dependent mode on the 15 most intense peaks by the linear ion trap. The MS2 isolation window as 2.0 m*/z* wide and the normalized collision energy was 35%. The precursor ions were selected based on charge states (+2) and intensity thresholds (above 1e5) from the MS1 scan; dynamic exclusion (one repeat during 30 s, a 60-s exclusion time window, 15 ppm tolerance) was also used. The MS proteomics data have been deposited to the ProteomeXchange Consortium via the PRIDE partner repository with the dataset identifier PXD056971.

### MS data processing

Raw MS data files were processed by MaxQuant [version 2.4.2.0; (Tyanova et al., 2016)] using the UniProtKB *Canus lupis familiaris* canonical sequence database (downloaded December 2023) and the human Afadin entry [UniProt Accession O35889 (UniProt Consortium, 2019)]. The following parameters were used: specific tryptic digestion with up to two missed cleavages, fixed carbamidomethyl modification, variable modifications for protein N-terminal acetylation, methionine oxidation, lysine biotinylation, match between runs, and label-free quantification. Prey proteins were filtered for high-confidence physical interactions and proximal proteins by scoring with SAINTexpress (v3.6.3) (Teo et al., 2014). SAINT was executed three separate times to score the following conditions: (1) Afadin-BL versus control, (2) BL-Afadin versus control, and (3) Afadin in ZOdKD cells versus control. A SAINT threshold of AvgP≥0.9 was used in all cases.

The following procedure was used to compute fold-changes. First, log2 imputed intensities were exponentiated, then averages were computed for each condition of interest, followed by computing a log2 fold-change between two conditions, and lastly the fold-changes were normalized to the bait by subtracting by the AF6 log2 fold-change. For example, for the AF6-N versus AF6-N ZO-KD comparison, the two AF6-N replicates were averaged and compared to the AF6-N ZO-KD experiment, while for the AF6 versus AF6 ZO-KD comparison, the two AF6-N and two AF6-C replicates were averaged and compared to the average of AF6-N ZO-KD and AF6-C ZO-KD. Due to the limited number of replicates, statistical confidence of the changes could not be computed.

The mass spectrometry proteomics data have been deposited to the ProteomeXchange Consortium via the PRIDE [1] partner repository with the dataset identifier PXD056971.

### Detailed author contributions

Wangsun Choi, Alan Fanning and Mark Peifer conceived the project with advice from Ben Major and Dennis Goldfarb. Wangsun Choi carried out all of the cell biological work, up to preparing the samples for mass spectroscopy. Feng Yan and Ben Major carried out the mass spectroscopy and Dennis Goldfarb analyzed the mass spectroscopy data. Mark Peifer, Ben Major and Dennis Goldfarb wrote the paper with editorial contributions from the other authors.

## Supplementary Material

10.1242/biolopen.061811_sup1 Supplementary information

Table S1.

Table S2. Overlap of our lists with BioGRID*

Table S3. GO Terms enriched in BL-Afadin list

Table S4. GO Terms enriched in Afadin-BL list

Table S5. Differential enrichment of proteins identified in parental MDCK cells versus ZOdKD MDCK Cells

Table S6. Matches with other Screens (Listed alphabetically)
