# Supplementary material for: Proximity proteomics provides a new resource for exploring the function of Afadin and the complexity of cell-cell adherens junctions
Source: Biol Open. 2025 Jan 30;14(2):bio061811. doi: 10.1242/bio.061811 (PMC11810119; doi:10.1242/bio.061811)
Supplement: Supplementary information [file biolopen-14-061811-s1.pdf]

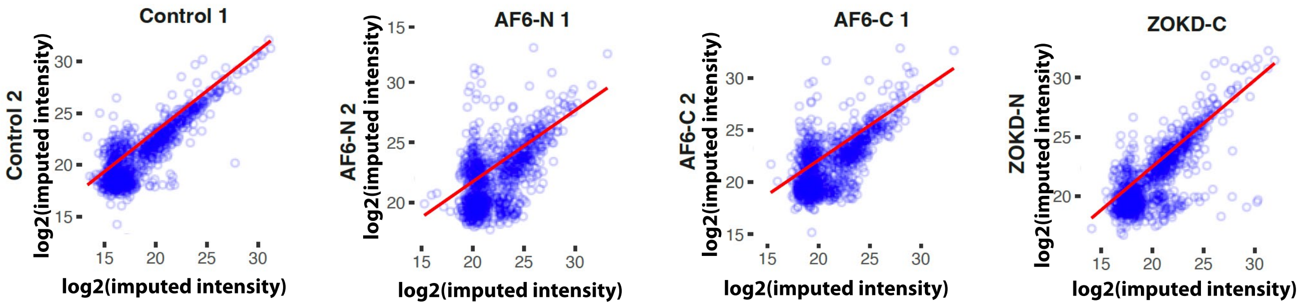

Fig. S1. Pairwise correlation plot examining variance between replicates.

Table1 and Table 2 References confirming junctional localization or cytoskeletal function

1. TJP1 AJs/TJs (Itoh et al., 1993)
2. SORBS2 AJs/Desmosomes (Fredriksson-Lidman et al., 2017) (Ding et al., 2020)
3. TJP2 AJs/TJs (Jesaitis and Goodenough, 1994)
4. LMO7 AJs (Ooshio et al., 2004)
5. ERBIN AJs (Choi et al., 2019)
6. TJP3 AJs/TJs (Haskins et al., 1998)
7. SCRIB AJs (Bonello et al., 2019)
8. LPP AJs (Van Itallie et al., 2014)
9. TP53BP2 AJs (Royer et al., 2022)
10. CGN TJs (Citi et al., 1988)
11. PARD3B AJs/TJs (Kohjima et al., 2002)
12. CTNND1 AJs (Reynolds et al., 1992)
13. ABLIM3 AJs (Matsuda et al., 2010)
14. SORBS1 AJs (Mandai et al., 1999)
15. PLEKHA6 AJs (Sluysmans et al., 2021b)
16. PLEKHA5 AJs (Sluysmans et al., 2021a)
17. CTNNA1 AJs (Nagafuchi et al., 1991)
18. NECTIN3 AJs (Satoh-Horikawa et al., 2000)
19. DBNL AJs (Herrera et al., 2021)
20. LIMA1 AJs (Abe and Takeichi, 2008)
21. SHROOM2 TJs(Tabaries et al., 2019)
22. PDLIM7 AJs, FAs (He et al., 2023) (Etournay et al., 2007)
23. CTTN AJs/Desmosomes (Moztarzadeh et al., 2024; Nekrasova et al., 2018)
24. PPL Desmosomes (Ruhrberg et al., 1997)
25, LASP1 AJs (Beckmann et al., 2021)
26. DLG1 AJs (Awadia et al., 2019)
27. NECTIN2 AJs (Miyahara et al., 2000)
28. DVL3 TJs (Diaz-Coranguez et al., 2023)
29. SHROOM3 AJs (Choi et al., 2016)
30. ZYXIN AJs (Bertocchi et al., 2017)
31. EPB41 TJs (Mattagajasingh et al., 2000)
32. RASSF8 AJs (Langton et al., 2009)
33. ARHGAP29 Apical to AJs (Tan et al., 2020)
34. TALLIN FAs (Horwitz et al., 1986)
35. PTPN11 AJs (Ukropec et al., 2000)
36. DIAPH2 AJs/TJs (Erasmus et al., 2016) (Higashi et al., 2019)
37. VCL AJs/FAs (Geiger et al., 1980) (Shriver and Rohrschneider, 1981)
38. PXN FAs (Turner et al., 1990)
39. SORBS3 FAs (Kioka et al., 1999)
40. CXADR AJs/TJs (Kwon et al., 2016)
41. CCDC85C AJs (Markham et al., 2014)
42. MAGI3 AJs/TJs (Adamsky et al., 2003)

Biology Open • Supplementary information

43. PAK4 AJs (Baskaran et al., 2021)
44. FNBP1L TJs (Van Itallie et al., 2015)
45. MAGI1 AJs (Yamada et al., 2004) (Mino et al., 2000)
46. PKP4 Desmosomes (Hatzfeld and Nachtsheim, 1996)
47. PAK2 AJs/TJs (Campbell et al., 2019) (Tornavaca et al., 2015)
48. PLEKHA7 AJs (Kurita et al., 2013)
49. FERMT2 FAs (Ma et al., 2008)
50. RSU1 FAs (Dougherty et al., 2008)
51. PARD3 AJs/TJs (Manabe et al., 2002) (Izumi et al., 1998)
52. NIBAN2 AJs (Chen et al., 2011)
53. PPP2R2A Desmosomes (Perl et al., 2023)

Abe, K. and Takeichi, M. (2008). EPLIN mediates linkage of the cadherin catenin complex to F-actin and stabilizes the circumferential actin belt. *Proc Natl Acad Sci U S A* **105**, 13-19.

Adamsky, K., Arnold, K., Sabanay, H. and Peles, E. (2003). Junctional protein MAGI-3 interacts with receptor tyrosine phosphatase beta (RPTP beta) and tyrosine-phosphorylated proteins. *J Cell Sci* **116**, 1279-1289.

Awadia, S., Huq, F., Arnold, T. R., Goicoechea, S. M., Sun, Y. J., Hou, T., Kreider-Letterman, G., Massimi, P., Banks, L., Fuentes, E. J., et al. (2019). SGEF forms a complex with Scribble and Dlg1 and regulates epithelial junctions and contractility. *J Cell Biol* **218**, 2699-2725.

Baskaran, Y., Tay, F. P., Ng, E. Y. W., Swa, C. L. F., Wee, S., Gunaratne, J. and Manser, E. (2021). Proximity proteomics identifies PAK4 as a component of Afadin-Nectin junctions. *Nat Commun* **12**, 5315.

Beckmann, D., Romer-Hillmann, A., Krause, A., Hansen, U., Wehmeyer, C., Intemann, J., de Gorter, D. J. J., Dankbar, B., Hillen, J., Heitzmann, M., et al. (2021). Lasp1 regulates adherens junction dynamics and fibroblast transformation in destructive arthritis. *Nat Commun* **12**, 3624.

Bertocchi, C., Wang, Y., Ravasio, A., Hara, Y., Wu, Y., Sailov, T., Baird, M. A., Davidson, M. W., Zaidel-Bar, R., Toyama, Y., et al. (2017). Nanoscale architecture of cadherin-based cell adhesions. *Nat Cell Biol* **19**, 28-37.

Bonello, T. T., Choi, W. and Peifer, M. (2019). Scribble and Discs-large direct initial assembly and positioning of adherens junctions during the establishment of apical-basal polarity. *Development* **146**.

Campbell, H. K., Salvi, A. M., O'Brien, T., Superfine, R. and DeMali, K. A. (2019). PAK2 links cell survival to mechanotransduction and metabolism. *J Cell Biol* **218**, 1958-1971.

Chen, S., Evans, H. G. and Evans, D. R. (2011). FAM129B/MINERVA, a novel adherens junction-associated protein, suppresses apoptosis in HeLa cells. *J Biol Chem* **286**, 10201-10209.

Biology Open • Supplementary information

Choi, J., Troyanovsky, R. B., Indra, I., Mitchell, B. J. and Troyanovsky, S. M. (2019). Scribble, Erbin, and Lano redundantly regulate epithelial polarity and apical adhesion complex. *J Cell Biol* **218**, 2277-2293.

Choi, W., Acharya, B. R., Peyret, G., Fardin, M. A., Mege, R. M., Ladoux, B., Yap, A. S., Fanning, A. S. and Peifer, M. (2016). Remodeling the zonula adherens in response to tension and the role of afadin in this response. *J Cell Biol* **213**, 243-260.

Citi, S., Sabanay, H., Jakes, R., Geiger, B. and Kendrick-Jones, J. (1988). Cingulin, a new peripheral component of tight junctions. *Nature* **333**, 272-276.

Diaz-Coranguez, M., Gonzalez-Gonzalez, L., Wang, A., Liu, X. and Antonetti, D. A. (2023). Disheveled-1 Interacts with Claudin-5 and Contributes to Norrin-Induced Endothelial Barrier Restoration. *Cells* **12**.

Ding, Y., Yang, J., Chen, P., Lu, T., Jiao, K., Tester, D. J., Giudicessi, J. R., Jiang, K., Ackerman, M. J., Li, Y., et al. (2020). Knockout of SORBS2 Protein Disrupts the Structural Integrity of Intercalated Disc and Manifests Features of Arrhythmogenic Cardiomyopathy. *J Am Heart Assoc* **9**, e017055.

Dougherty, G. W., Jose, C., Gimona, M. and Cutler, M. L. (2008). The Rsu-1-PINCH1-ILK complex is regulated by Ras activation in tumor cells. *Eur J Cell Biol* **87**, 721-734.

Erasmus, J. C., Bruche, S., Pizarro, L., Maimari, N., Pogglioli, T., Tomlinson, C., Lees, J., Zalivina, I., Wheeler, A., Alberts, A., et al. (2016). Defining functional interactions during biogenesis of epithelial junctions. *Nat Commun* **7**, 13542.

Etournay, R., Zwaenepoel, I., Perfettini, I., Legrain, P., Petit, C. and El-Amraoui, A. (2007). Shroom2, a myosin-VIIa- and actin-binding protein, directly interacts with ZO-1 at tight junctions. *J Cell Sci* **120**, 2838-2850.

Fredriksson-Lidman, K., Van Itallie, C. M., Tietgens, A. J. and Anderson, J. M. (2017). Sorbin and SH3 domain-containing protein 2 (SORBS2) is a component of the acto-myosin ring at the apical junctional complex in epithelial cells. *PLoS One* **12**, e0185448.

Geiger, B., Tokuyasu, K. T., Dutton, A. H. and Singer, S. J. (1980). Vinculin, an intracellular protein localized at specialized sites where microfilament bundles terminate at cell membranes. *Proc Natl Acad Sci U S A* **77**, 4127-4131.

Haskins, J., Gu, L., Wittchen, E. S., Hibbard, J. and Stevenson, B. R. (1998). ZO-3, a novel member of the MAGUK protein family found at the tight junction, interacts with ZO-1 and occludin. *J Cell Biol* **141**, 199-208.

Hatzfeld, M. and Nachtsheim, C. (1996). Cloning and characterization of a new armadillo family member, p0071, associated with the junctional plaque: evidence for a subfamily of closely related proteins. *Journal of Cell Science* **109**, 2767-2778.

He, Q., Sze, S. K., Ng, K. S. and Koh, C. G. (2023). Paxillin interactome identified by SILAC and label-free approaches coupled to TurboID sheds light on the compositions of focal adhesions in mouse embryonic stem cells. *Biochem Biophys Res Commun* **680**, 73-85.

Herrera, A., Menendez, A., Torroba, B., Ochoa, A. and Pons, S. (2021). Dbnl and beta-catenin promote pro-N-cadherin processing to maintain apico-basal polarity. *J Cell Biol* **220**.

Higashi, T., Stephenson, R. E. and Miller, A. L. (2019). Comprehensive analysis of formin localization in Xenopus epithelial cells. *Mol Biol Cell* **30**, 82-95.

Horwitz, A., Duggan, K., Buck, C., Beckerle, M. C. and Burridge, K. (1986). Interaction of plasma membrane fibronectin receptor with talin--a transmembrane linkage. *Nature* **320**, 531-533.

Itoh, M., Nagafuchi, A., Yonemura, S., Kitani-Yasuda, T., Tsukita, S. and Tsukita, S. (1993). The 220-kD protein colocalizing with cadherins in non-epithelial cells is identical to ZO-1, a tight junction-associated protein in epithelial cells: cDNA cloning and immunoelectron microscopy. *Journal of Cell Biology* **121**, 491-502.

Izumi, Y., Hirose, T., Tamai, Y., Hirai, S., Nagashima, Y., Fujimoto, T., Tabuse, Y., Kemphues, K. and Ohno, S. (1998). An atypical PKC directly associates and colocalizes at the epithelial tight junction with ASIP, a mammalian homologue of Caenorhabditis elegans polarity protein PAR-3. *Journal of Cell Biology* **143**, 95-106.

Jesaitis, L. A. and Goodenough, D. A. (1994). Molecular characterization and tissue distribution of ZO-2, a tight junction protein homologous to ZO-1 and the Drosophila discs-large tumor suppressor protein. *J Cell Biol* **124**, 949-961.

Kioka, N., Sakata, S., Kawauchi, T., Amachi, T., Akiyama, S. K., Okazaki, K., Yaen, C., Yamada, K. M. and Aota, S. (1999). Vinexin: a novel vinculin-binding protein with multiple SH3 domains enhances actin cytoskeletal organization. *J Cell Biol* **144**, 59-69.

Kohjima, M., Noda, Y., Takeya, R., Saito, N., Takeuchi, K. and Sumimoto, H. (2002). PAR3beta, a novel homologue of the cell polarity protein PAR3, localizes to tight junctions. *Biochem Biophys Res Commun* **299**, 641-646.

Kurita, S., Yamada, T., Rikitsu, E., Ikeda, W. and Takai, Y. (2013). Binding between the junctional proteins afadin and PLEKHA7 and implication in the formation of adherens junction in epithelial cells. *J Biol Chem* **288**, 29356-29368.

Kwon, J. W., Kim, N. H. and Choi, I. (2016). CXADR is required for AJ and TJ assembly during porcine blastocyst formation. *Reproduction* **151**, 297-304.

Langton, P. F., Colombani, J., Chan, E. H., Wepf, A., Gstaiger, M. and Tapon, N. (2009). The dASPP-dRASSF8 complex regulates cell-cell adhesion during Drosophila retinal morphogenesis. *Curr Biol* **19**, 1969-1978.

Ma, Y. Q., Qin, J., Wu, C. and Plow, E. F. (2008). Kindlin-2 (Mig-2): a co-activator of beta3 integrins. *J Cell Biol* **181**, 439-446.

Manabe, N., Hirai, S., Imai, F., Nakanishi, H., Takai, Y. and Ohno, S. (2002). Association of ASIP/mPAR-3 with adherens junctions of mouse neuroepithelial cells. *Dev. Dyn.* **225**, 61-69.

Mandai, K., Nakanishi, H., Satoh, A., Takahashi, K., Satoh, K., Nishioka, H., Mizoguchi, A. and Takai, Y. (1999). Ponsin/SH3P12: an l-afadin- and vinculin-binding protein localized at cell-cell and cell-matrix adherens junctions. *J Cell Biol* **144**, 1001-1017.

Markham, N. O., Doll, C. A., Dohn, M. R., Miller, R. K., Yu, H., Coffey, R. J., McCrea, P. D., Gamse, J. T. and Reynolds, A. B. (2014). DIPA-family coiled-coils bind conserved isoform-specific head domain of p120-catenin family: potential roles in hydrocephalus and heterotopia. *Mol Biol Cell* **25**, 2592-2603.

Matsuda, M., Yamashita, J. K., Tsukita, S. and Furuse, M. (2010). abLIM3 is a novel component of adherens junctions with actin-binding activity. *Eur J Cell Biol* **89**, 807-816.

Mattagajasingh, S. N., Huang, S. C., Hartenstein, J. S. and Benz, E. J., Jr. (2000). Characterization of the interaction between protein 4.1R and ZO-2. A possible link between the tight junction and the actin cytoskeleton. *J Biol Chem* **275**, 30573-30585.

Mino, A., Ohtsuka, T., Inoue, E. and Takai, Y. (2000). Membrane-associated guanylate kinase with inverted orientation (MAGI)-1/brain angiogenesis inhibitor 1-associated protein (BAP1) as a scaffolding molecule for Rap small G protein GDP/GTP exchange protein at tight junctions. *Genes Cells* **5**, 1009-1016.

Miyahara, M., Nakanishi, H., Takahashi, K., Satoh-Horikawa, K., Tachibana, K. and Takai, Y. (2000). Interaction of nectin with afadin is necessary for its clustering at cell-cell contact sites but not for its cis dimerization or trans interaction. *J Biol Chem* **275**, 613-618.

Moztarzadeh, S., Sepic, S., Hamad, I., Waschke, J., Radeva, M. Y. and Garcia-Ponce, A. (2024). Cortactin is in a complex with VE-cadherin and is required for endothelial adherens junction stability through Rap1/Rac1 activation. *Scientific reports* **14**, 1218.

Nagafuchi, A., Takeichi, M. and Tsukita, S. (1991). The 102 kd cadherin-associated protein: similarity to vinculin and posttranscriptional regulation of expression. *Cell* **65**, 849-857.

Nekrasova, O., Harmon, R. M., Broussard, J. A., Koetsier, J. L., Godsel, L. M., Fitz, G. N., Gardel, M. L. and Green, K. J. (2018). Desmosomal cadherin association with Tctex-1 and cortactin-Arp2/3 drives perijunctional actin polymerization to promote keratinocyte delamination. *Nat Commun* **9**, 1053.

Ooshio, T., Irie, K., Morimoto, K., Fukuhara, A., Imai, T. and Takai, Y. (2004). Involvement of LMO7 in the association of two cell-cell adhesion molecules, nectin and E-cadherin, through afadin and alpha-actinin in epithelial cells. *J Biol Chem* **279**, 31365-31373.

Perl, A. L., Koetsier, J. L. and Green, K. J. (2023). PP2A-B55alpha controls keratinocyte adhesion through dephosphorylation of the Desmoplakin C-terminus. *Scientific reports* **13**, 12720.

Reynolds, A. B., Herbert, L., Cleveland, J. L., Berg, S. T. and Gaut, J. R. (1992). p120, a novel substrate of protein tyrosine kinase receptors and of p60v-src, is related to cadherin binding factors ß-catenin, plakoglobin, and *armadillo*. *Oncogene* **7**, 2439-2445.

Royer, C., Sandham, E., Slee, E., Schneider, F., Lagerholm, C. B., Godwin, J., Veits, N., Hathrell, H., Zhou, F., Leonavicius, K., et al. (2022). ASPP2 maintains the integrity of mechanically stressed pseudostratified epithelia during morphogenesis. *Nat Commun* **13**, 941.

Ruhrberg, C., Hajibagheri, M. A., Parry, D. A. and Watt, F. M. (1997). Periplakin, a novel component of cornified envelopes and desmosomes that belongs to the plakin family and forms complexes with envoplakin. *J Cell Biol* **139**, 1835-1849.

Satoh-Horikawa, K., Nakanishi, H., Takahashi, K., Miyahara, M., Nishimura, M., Tachibana, K., Mizoguchi, A. and Takai, Y. (2000). Nectin-3, a new member of immunoglobulin-like cell adhesion molecules that shows homophilic and heterophilic cell-cell adhesion activities. *J Biol Chem* **275**, 10291-10299.

Shriver, K. and Rohrschneider, L. (1981). Organization of pp60src and selected cytoskeletal proteins within adhesion plaques and junctions of Rous sarcoma virus-transformed rat cells. *J Cell Biol* **89**, 525-535.

Sluysmans, S., Mean, I., Jond, L. and Citi, S. (2021a). WW, PH and C-Terminal Domains Cooperate to Direct the Subcellular Localizations of PLEKHA5, PLEKHA6 and PLEKHA7. *Front Cell Dev Biol* **9**, 729444.

Sluysmans, S., Mean, I., Xiao, T., Boukhatemi, A., Ferreira, F., Jond, L., Mutero, A., Chang, C. J. and Citi, S. (2021b). PLEKHA5, PLEKHA6, and PLEKHA7 bind to PDZD11 to target the Menkes ATPase ATP7A to the cell periphery and regulate copper homeostasis. *Mol Biol Cell* **32**, ar34.

Tabaries, S., McNulty, A., Ouellet, V., Annis, M. G., Dessureault, M., Vinette, M., Hachem, Y., Lavoie, B., Omeroglu, A., Simon, H. G., et al. (2019). Afadin cooperates with Claudin-2 to promote breast cancer metastasis. *Genes Dev* **33**, 180-193.

Tan, B., Yatim, S., Peng, S., Gunaratne, J., Hunziker, W. and Ludwig, A. (2020). The Mammalian Crumbs Complex Defines a Distinct Polarity Domain Apical of Epithelial Tight Junctions. *Curr Biol* **30**, 2791-2804 e2796.

Tornavaca, O., Chia, M., Dufton, N., Almagro, L. O., Conway, D. E., Randi, A. M., Schwartz, M. A., Matter, K. and Balda, M. S. (2015). ZO-1 controls endothelial adherens junctions, cell-cell tension, angiogenesis, and barrier formation. *J Cell Biol* **208**, 821-838.

Turner, C. E., Glenney, J. R., Jr. and Burridge, K. (1990). Paxillin: a new vinculin-binding protein present in focal adhesions. *J Cell Biol* **111**, 1059-1068.

Ukropec, J. A., Hollinger, M. K., Salva, S. M. and Woolkalis, M. J. (2000). SHP2 association with VE-cadherin complexes in human endothelial cells is regulated by thrombin. *J Biol Chem* **275**, 5983-5986.

Van Itallie, C. M., Tietgens, A. J., Aponte, A., Fredriksson, K., Fanning, A. S., Gucek, M. and Anderson, J. M. (2014). Biotin ligase tagging identifies proteins proximal to E-cadherin, including lipoma preferred partner, a regulator of epithelial cell-cell and cell-substrate adhesion. *J Cell Sci* **127**, 885-895.

Van Itallie, C. M., Tietgens, A. J., Krystofiak, E., Kachar, B. and Anderson, J. M. (2015). A complex of ZO-1 and the BAR-domain protein TOCA-1 regulates actin assembly at the tight junction. *Mol Biol Cell* **26**, 2769-2787.

Yamada, A., Irie, K., Fukuhara, A., Ooshio, T. and Takai, Y. (2004). Requirement of the actin cytoskeleton for the association of nectins with other cell adhesion molecules at adherens and tight junctions in MDCK cells. *Genes Cells* **9**, 843-855.

Table S1.

Available for download at
https://journals.biologists.com/bio/article-lookup/doi/10.1242/bio.061811#supplementary-data

Table S2. Overlap of our lists with BioGRID*

Available for download at
https://journals.biologists.com/bio/article-lookup/doi/10.1242/bio.061811#supplementary-data

Table S3. GO Terms enriched in BL-Afadin list

Available for download at
https://journals.biologists.com/bio/article-lookup/doi/10.1242/bio.061811#supplementary-data

Table S4. GO Terms enriched in Afadin-BL list

Available for download at
https://journals.biologists.com/bio/article-lookup/doi/10.1242/bio.061811#supplementary-data

Table S5. Differential enrichment of proteins identified in parental MDCK cells versus ZOdKD MDCK Cells

Available for download at
https://journals.biologists.com/bio/article-lookup/doi/10.1242/bio.061811#supplementary-data

Table S6. Matches with other Screens (Listed alphabetically)

Available for download at
https://journals.biologists.com/bio/article-lookup/doi/10.1242/bio.061811#supplementary-data
